# Synthesis and Characterization of Two-Dimensional Conjugated Polymers Incorporating Electron-Deficient Moieties for Application in Organic Photovoltaics

**DOI:** 10.3390/polym8110382

**Published:** 2016-10-27

**Authors:** Chuen-Yo Hsiow, Han-Ying Wang, Yu-Hsiang Lin, Rathinam Raja, Syang-Peng Rwei, Wen-Yen Chiu, Chi-An Dai, Leeyih Wang

**Affiliations:** 1 Center for Condensed Matter Sciences, National Taiwan University, Taipei 10617, Taiwan; rajaorgchem80@gmail.com; 2 Joint Center for Artificial Photosynthesis, California Institute of Technology, Pasadena, CA 91125, USA; 3 Institute of Organic and Polymeric Materials, National Taipei University of Technology, Taipei 10608, Taiwan; hanying1989@gmail.com (H.-Y.W.); sean76326@hotmail.com (Y.-H.L.); f10714@ntut.edu.tw (S.-P.R.); 4 Department of Chemical Engineering, National Taiwan University, Taipei 10617, Taiwan; ycchiu@ntu.edu.tw (W.-Y.C.); polymer@ntu.edu.tw (C.-A.D.)

**Keywords:** two-dimensional conjugated polymer, polymer solar cell, benzo[1,2-b:4,5-b′]dithiophene, thieno[3,4-*c*]pyrrole-4,6-dione, pyrrolo[3,4-*c*]pyrrole-1,4-dione, conjugated side chain.

## Abstract

A series of novel p-type conjugated copolymers, PTTVBDT, PTTVBDT-TPD, and PTTVBDT-DPP, cooperating benzo[1,2-b:4,5-b′]dithiophene (BDT) and terthiophene-vinylene (TTV) units with/without thieno[3,4-*c*]pyrrole-4,6-dione (TPD) or pyrrolo[3,4-*c*]pyrrole-1,4-dione (DPP) via Stille polymerization were synthesized and characterized. Copolymer PTTVBDT shows a low-lying HOMO energy level and ordered molecular-packing behavior. Furthermore, two terpolymers, PTTVBDT-TPD and PTTVBDT-DPP, display stronger absorption ability, alower-lying HOMO energy level, and preferred molecular orientation, due to the replacement TTV-monomer units with electron-deficient groups. Furthermore, bulk-heterojunction organic solar cells were fabricated using blends of the PTTVBDT-TPD, and PC_61_BM gave the best power conversion efficiency of 5.01% under the illumination of AM 1.5G, 100 mW·cm^−2^; the short circuit current (*J*_sc_) was 11.65 mA·cm^−2^ which displayed a 43.8% improvement in comparison with the PTTVBDT/PC_61_BM device. These results demonstrate a valid strategy combining the two-dimensional molecular structure with random copolymerization strikes promising conjugated polymers to achieve highly efficient organic photovoltaics.

## 1. Introduction

Development of novel semiconducting conjugated polymers and investigation ofstructure–property relationships always play a critical role in the organic electronic devices. Through rational molecular design, they will be tuned specifically to achieve desired optical and electronic properties for application in electronics and optoelectronics, such as field effect transistors (FETs) [1,2], photo-detectors [3], light-emitting diodes (LEDs) [4], and photovoltaics (PVs) [5,6]. Over the past several decades, many well-designed conjugated polymers have been used as photoactive materials in organic photovoltaic (OPV) devices owing to their excellent optoelectronic properties [7,8]. As a promising conjugated polymer in the OPV field, it should possess broad absorption in the solar spectrum, high charge carrier mobility for charge transport, suitable energy levels, etc. [7,9]. Therefore, how the designs of the molecular structures can be used to control their properties is of particular importance in the development of conjugated polymers.

**Figure 1:**
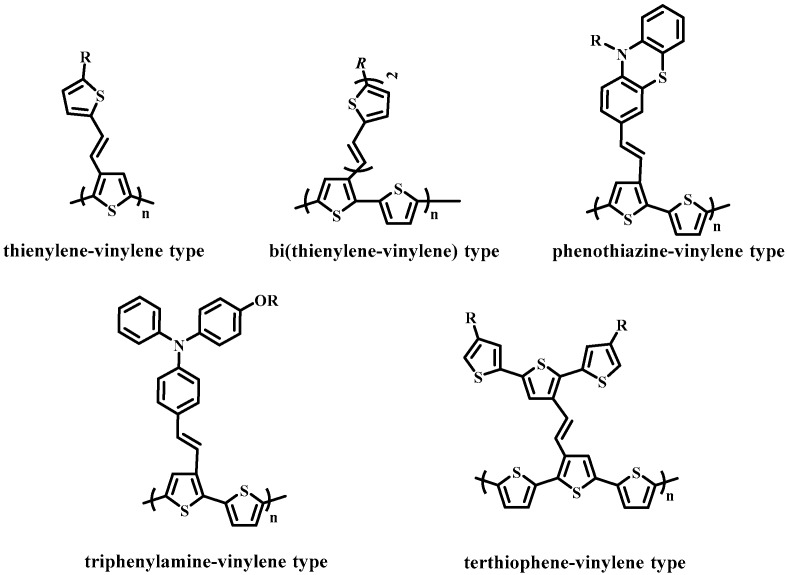
Molecular structures of the 2-D conjugated polythiophene derivatives (R = alkyl group) [16,17,18,19,20].

Among these high performance conjugated polymers, benzo[1,2-b:4,5-b′]dithiophene (BDT) has been considered as one of the most effective building-blocks for p-type conjugated polymers because it possesses a planar structure and BDT-based copolymers have high hole mobility and suitable electronic energy levels [10,11,12]. The bulk heterojunction (BHJ) polymer solar cell devices (PSCs) based on BDT-based copolymers have achieved high power conversion efficiency (PCE) values over 10% [8,13,14,15]. In addition, in order to enhance the short-circuit current density in PSCs, molecular designs should be conducive to enhancing the absorption ability of the conjugated polymer. As shown in Figure 1, two-dimensional (2-D) conjugation by vinylene linkage, such as thienylene-vinylene [16], bi(thienylene-vinylene) [17], phenothiazine-vinylene [18], triphenylamine-vinylene [19], and terthiophene-vinylene [20], has proven to be a successful strategy to enhance light-harvesting ability, charge mobility and self-assembly, and adjust molecular energy levels to meet the requirements of PSCs [17,20,21]. Moreover, Tan and co-workers reported that a series of 2-D conjugated polymers incorporating different thienylene-vinylene derivatives with BDT building blocks, such as PTG1 [22] and PBDT-TID [23], not only attracted much interest but also demonstrated an effective strategy to extend the absorption range and down-shift the HOMO energy level. On the other hand, Li and co-workers reported that introduction of strong electro-withdrawing groups, such as pyrrolo[3,4-*c*]pyrrole-1,4-dione (DPP), in the 2-D conjugated polymers broadens the spectral absorption via the random polymerization [24]. Taking a comprehensive view of these strategies, it it is of great interest to discover novel 2-D conjugated polymers to improve their optoelectronic and photovolatic properties, such as enhancing absorption ability and further improving photon-to-electron conversion efficiency, through incorporating different 2-D conjugation blocks with electron-withdrawing and/or electron-donating groups.

**Figure 2:**
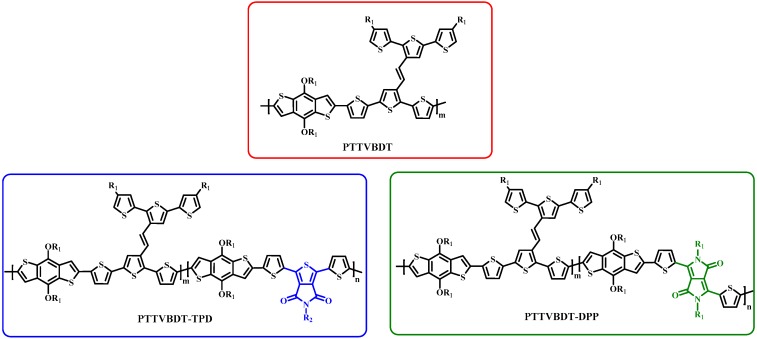
Chemical Structures of PTTVBDT, PTTVBDT-TPD, and PTTVBDT-DPP (R_1_ = 2-ethylhexyl; R_2_ = *n*-octyl).

In our previous studies, terthiophene–vinylene (TTV)-based conjugated polymers possess a broader absorption region and deeper HOMO levels than rr-P3HT [20,25,26]. Herein, a novel 2-D polymer PTTVBDT, as shown in Figure 2, has been designed and synthesized by cooperating TTV and BDT units via Stille polymerization. Furthermore, two electron-withdrawing groups, TPD and DPP units, were employed as the third copolymerization unit to build the terpolymers, PTTVBDT-TPD and PTTVBDT-DPP, respectively. Thieno[3,4-*c*]pyrrole-4,6-dione (TPD) and DPP units both have been also widely used as building blocks and electron-withdrawing groups in high performance conjugated polymers due to their symmetric, rigidly fused, and coplanar structures [11,27,28,29,30]. For these three polymers, their optical, thermal, molecular energy levels, crystallographic, and photovoltaic properties were characterized carefully and systematically, making comparisons between each of them. The best PSCs based on PTTVBDT-TPD/PC_61_BM gave a PCE of 5.01% with monochromatic incident photon-to-electron conversion efficiency (IPCE) >60% over the range of 340–600 nm under AM 1.5G illumination with an intensity of 100 mW·cm^−2^.

## 2. Experimental Section

### 2.1. Materials

All reagents were purchased from Acros (purchased from Dinhaw Enterprise Co., New Taipei city, Taiwan), Aldrich (purchased from UNI-ONWARD Co., New Taipei city, Taiwan), or Alfa Aesar (purchased from ECHO Chemical Co., Taipei city, Taiwan) and used without further purification unless otherwise noted. 2,6-Bis(trimethyltin)-4,8-bis(2-ethylhexyloxy)benzo[1,2-b:4,5-b′]dithiophene (M2) [31] and 3,6-bis(5-bromothiophen-2-yl)-2,5-bis(2-ethylhexyl)pyrrolo[3,4-*c*]pyrrole-1,4(2*H*,5*H*)-dione (M3) [32] were prepared via literature procedures. Poly(3,4-ethylenedioxythiophene):poly-(styrenesulfonate) (PEDOT:PSS, Bytron P AI4083) was purchased from HC Starck (Berlin, Germany).

### 2.2. Instrument and Characterization

^1^H NMR and ^13^C NMR spectra were recorded in chloroform-*d* (CDCl_3_) solution at 400 MHz on Bruker DRX-400 spectrometer (Bruker Taiwan Co., Taipei, Taiwan). All NMR spectra were calibrated by CDCl_3_ where ^1^H NMR chemical shifts of CDCl_3_ is 7.23 ppm; in addition, ^13^CNMR of CDCl_3_ is 77.0 ppm. Microwave assisted Stille cross-coupling reactions were performed in Anton Paar Monowave 300 microwave reactor (Graz, Austria). Ultraviolet–visible (UV–vis) absorption spectra in solution or solid thin film were recorded on a JASCO MD-2010 spectrometer (JASCO, Tokyo, Japan). Gel permeation chromatography (GPC) was conducted at 40 °C using two Jordi DVB mixed-bed columns (250 mm (length) × 10 mm (inner diameter, ID); suitable for separating polymers with molecular weights from 1 × 10^2^ to 1 × 10^7^ g·mol^−1^) using THF as the eluent at a flow rate of 1.0 mL/min on a JASCO instrument (JASCO, Tokyo, Japan) that was equipped with UV–vis and refractive index (RI) detectors connected in series. The work functions of materials were measured using an AC2 photoelectron spectrometer (Riken Keiki Co., Tokyo, Japan). Atomic force microscopy (AFM) images were captured using taping model in Digital Instruments Nanoscope III (Eugene, OR, USA). The AFM samples were prepared by spin-coating the blend solution on a ITO/PEDOT:PSS substrate. The two-dimensional grazing incident X-ray diffraction (2D-GIXRD) samples were prepared by drop-casting from CB solution (10 g/L) on 10 mm × 10 mm Si wafers substrate and then thermal annealing at 120 °C, 20 min. 2D-GIXRD measurements were carried out on the wiggler beamline BL17A1 of the National Synchrotron Radiation Research Center (NSRRC, Hsinchu, Taiwan), with a wavelength of 1.333 Å delivered from a superconducting wavelength-shifting magnet and a Si (111) triangular crystal monochromator (TCM) [26,33]. The data were recorded by a Mar3450 image plate with exposure duration 60 s. The two-dimensional diffraction pattern was converted to a one-dimensional powder diffraction profile by fit-2D program. Furthermore, the diffraction patterns are fitted into the one-dimensional powder diffraction spectra using Gauss function of OriginPro 8 (Northampton, MA, USA) to locate the peak positions and calculate the full-width at half-maximum.

### 2.3. Device Fabrication and Characterization

The polymer solar cells (PSCs) were fabricated with the structure of ITO/PEDOT:PSS/polymer:PC_61_BM/Ca/Al from our previous work [20,26]. The ITO glasses were cleaned by a sequential ultrasonic treatment in detergent, deionized water, acetone, and isopropanol for 20 min. Then PEDOT:PSS was filtered through a 0.2-μm filter and spin-coated at 3500 rpm for 30 s on top of ITO electrode. Subsequently, the PEDOT:PSS film was baked at 140 °C for 10 min in the air, and then moved into a glovebox. The blend solution of PC_61_BM and synthesized polymers in a solvent was filtered with/without a 0.45-μm filter and spin-coated at 800 rpm for 30 s on top of the PEDOT:PSS layer. These devices were thermally annealed at various temperatures for 10 min, followed by capping with Ca (~20 nm) and then Al (~60 nm) in a thermal evaporator at a base pressure of ca. 10^±6^ Pa. The active area of the devices is 0.06 cm^2^. The current density–voltage (*J*–*V*) measurements of the devices were conducted on a computer-controlled Keithley 2400 Source Measure Unit under AM 1.5G simulated solar irradiation at 100 mW·cm^−2^. The light incident intensity was calibrated by a mono-Si reference cell with a KG5 filter (PV Measurements, Inc., Boulder, CO, USA), which was pre-calibrated by the National Renewable Energy Laboratory. The space charge limited currents (SCLC) measurements were carried out using a Keithley 2400 source meter (Keithley, Cleveland, OH, USA) under dark condition. The hole mobilities of were evaluated in a hole-only device configuration, ITO/PEDOT:PSS/(pristine polymer thin films or blend with PC_61_BM) (~200 nm)/Au (~80 nm); the electron mobilities of were evaluated in a device configuration, ITO/SAM-NH_2_ (self-assembled monolayer, 4-aminobenzoic acid)/(pristine polymer thin films or blend with PC_61_BM)/Ca (~20 nm)/Al (~60 nm). The IPCE spectra were recorded under illumination by a xenon lamp and a monochromator (TRIAX 180, JOBIN YVON, Edison, NJ, USA), and the light intensity was calibrated by using an OPHIR 2A-SH thermopile detector (OPHIR Photonics, North Logan, UT, USA).

### 2.4. Synthesis of Monomers and Polymers

#### 2.4.1. (*E*)-3′-(2-(5,5″-dibromo-[2,2′:5′,2″-terthiophen]-3′-yl)vinyl)-4,4″-bis(2-ethylhexyl)-2,2′:5′,2″-terthiophene (M1)

(*E*)-3′-(2-(5,5″-dibromo-[2,2′:5′,2″-terthiophen]-3′-yl)vinyl)-4,4″-bis(2-ethylhexyl)-2,2′:5′,2″-terthiophene (M1). Compound 1 (1.9 g, 3.2 mmol) was taken in a 100 mL Schlenk flask and degassed via vacuum-nitrogen cycle by three times. 20 mL of anhydrous THF was added and stirred in an ice bath for several minutes and then added with sodium *tert*-butoxide (0.33 g, 3.4 mmol). After stirring for 30 min, Compound 2 (1.37 g, 2.7 mmol) which was dissolved in anhydrous THF (10 mL) was added. The reaction mixture was warmed to room temperature and stirred overnight. The solution was poured into ammonium chloride aqueous solution and extracted twice with 40 mL ethyl acetate. The organic phase was combined and dried with MgSO_4_. After removal of the solvent, the residue was purified by flash column chromatography (eluent: Hexane, *R*_f_ = 0.4), yielding a yellow liquid M1 (1.3 g, 1.5 mmol, 56%) ^1^H NMR (500 MHz, CDCl_3_) δ 7.24 (s, 1H), 7.23 (d, *J* = 15.8 Hz, 1H), 7.20 (s, 1H), 7.07 (d, *J* = 15.8 Hz, 1H), 7.05 (d, *J* = 3.8 Hz, 1H), 7.01 (s, 1H), 6.98 (s, 1H), 6.95 (s, 1H), 6.94 (d, *J* = 3.8 Hz, 1H), 6.90 (d, *J* = 3.8 Hz, 1H), 6.88 (d, *J* = 3.8 Hz, 1H), 6.79 (s, 1H), 2.61–2.51 (m, 4H), 1.62–1.56 (m, 2H), 1.40–1.26 (m, 16H), 0.92–0.87 (m, 12H); ^13^C NMR (125 MHz, CDCl_3_) δ 142.92, 142.81, 137.88, 137.04, 136.46, 136.27, 136.98, 135.63, 135.32, 134.54, 132.47, 130.69, 130.67, 130.38, 128.73, 127.18, 125.99, 124.42, 124.32, 122.46, 122.22, 122.14, 121.64, 120.44, 113.26, 111.70, 40.34, 40.22, 34.55, 34.52, 32.55, 32.46, 28.90, 28.86, 25.65, 25.60, 23.04, 14.14, 10.87, 10.83.

#### 2.4.2. 5-Octyl-1,3-di(thiophen-2-yl)-4*H*-thieno[3,4-*c*]pyrrole-4,6(5*H*)-dione (4)

5-Octyl-1,3-di(thiophen-2-yl)-4*H*-thieno[3,4-*c*]pyrrole-4,6(5*H*)-dione (4). Compound 3 (1.0 g, 3 mmol), tributyl(thiophen-2-yl)stannane (2.8 g, 7.5 mmol), and Pd(PPh_3_)_4_ (35 mg, 0.03 mmol) were weighted into microwave tubes (G30) and then 12 mL anhydrous toluene were added. The tubes were directly subjected into the microwave reactor and processed the reaction conditions: (1) raise temperature from r.t. to 30 °C in 3 min; (2) raise temperature from 30 to 200 °C as fast as possible; (3) hold the temperature 90 min; cool down to 55 °C. The crude product was filtered with Celite to remove the metal catalyst. After removal of the solvent, the residue was purified by column chromatography (Hexane/chloroform = 4/1), yielding a yellow solid 4 (1.138 g, 2.61 mmol, 86%). ^1^H NMR (400 MHz, CDCl_3_) δ 7.98 (d, 2H, *J* = 3.6 Hz), 7.41 (d, 2H, *J* = 5.0 Hz), 7.10 (dd, 2H, *J* = 3.6, 5.0 Hz), 3.63 (t, 2H, *J* = 7.2 Hz), 1.65 (m, 2H), 1.23 (m, 10H), 0.84 (t, 3H, *J* = 6.6 Hz); ^13^C NMR (100 MHz, CDCl_3_) δ 162.51, 136.39, 132.41, 129.81, 128.56, 128.36, 38.54, 31.76, 29.67, 29.16, 28.46, 26.93, 22.59, 14.04.

#### 2.4.3. 1,3-Bis(5-bromothiophen-2-yl)-5-octyl-4*H*-thieno[3,4-*c*]pyrrole-4,6(5*H*)-dione (M3)

1,3-Bis(5-bromothiophen-2-yl)-5-octyl-4*H*-thieno[3,4-*c*]pyrrole-4,6(5*H*)-dione (M3). A mixture of 5 (0.432 g, 1.01 mmol) and *N*-bromosuccinimide (0.394 g, 2.2 mmol) in 50 mL chloroform and 50 mL acetic acid stirred overnight at room temperature. Then, the solution was poured into water and extracted twice with 50 mL chloroform. The organic phase was combined and dried with MgSO_4_. After removal of the solvent, the residue was purified by flash column chromatography (eluent: hexane/CH_2_Cl_2_ = 6/1), yielding yellow solid M3 (0.50 g, 0.85 mmol, 85%). ^1^H NMR (400 MHz, CDCl_3_) δ 7.63 (d, 2H, *J* = 4.0 Hz), 7.05 (d, 2H, *J* = 4.0 Hz), 3.61 (t, 2H, *J* = 7.2 Hz), 1.64 (m, 2H, *J* = 7.2 Hz), 1.22 (m, 10H) , 0.84 (t, 3H, *J* = 7.2 Hz); ^13^C NMR (100 MHz, CDCl_3_) δ 162.12, 134.90, 133.66, 131.05, 129.66, 128.48, 116.69, 38.61, 31.74, 29.66, 29.12, 28.40, 26.92, 22.559, 14.05.

#### 2.4.4. Standard Procedure of Polymerization and Purification

Monomers were first weighted into a 50 mL two neck round-bottom flask and then subjected to three successive cycles of vacuum followed by refilling with nitrogen. Then, Pd_2_dba_3_ (3 mol %)/P(*o*-tol)_3_ (6 mol %) were dissolved in anhydrous chlorobenzene and added into the reaction mixture via a syringe. The polymerization was carried out at 110 °C for 72 h. The raw product was dissolved by chloroform, then precipitated into methanol and collected through a Soxhlet thimble by filtration, which was then subjected to Soxhlet extraction with methanol, acetone, hexanes, methylene chloride and chloroform. The final polymer was recovered from chloroform fraction by rotary evaporation and dried in vacuum for 12 h at 60 °C.

#### 2.4.5. Synthesis of Polymers

PTTVBDT is synthesized according to the standard procedure with M1 (183 mg, 0.237 mmol) and M2 (214 mg, 0.237 mmol), yielding PTTVBDT (273 mg, 82%).

PTTVBDT-TPD is synthesized according to the standard procedure with M1 (167.4 mg, 0.174 mmol), M2 (168.4 mg, 0.218 mmol) and M3 (25.8 g, 0.044 mmol), yielding PTTVBDT-TPD (154.8 mg, 63.0%). Elemental analysis: found: S, 21.268%; H, 7.326%; C, 68.365%; N, 0.179%.

PTTVBDT-DPP is synthesized according to the standard procedure with M1 (178.9 mg, 0.198 mmol), M2 (191.5 mg, 0.248 mmol) and M4 (33.8 g, 0.049 mmol), yielding PTTVBDT-DPP (159.0 mg, 56%). Elemental analysis: found: S, 20.819%; H, 7.449%; C, 68.714%; N, 0.297%.

## 3. Results and Discussion

**Scheme 1:**
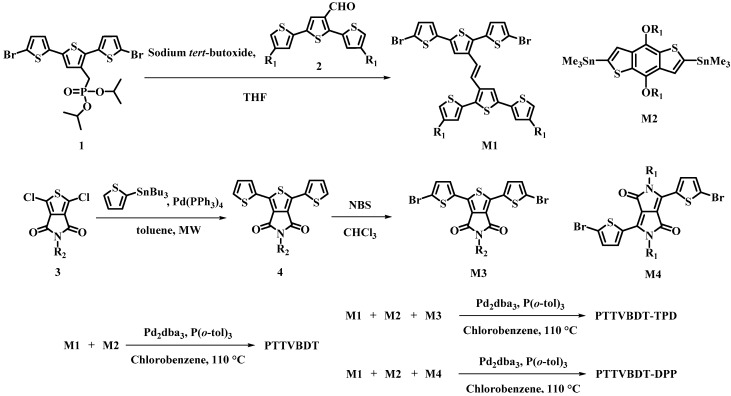
Synthetic Routes of the Monomers and the Corresponding Copolymers (R_1_ = 2-ethylhexyl; R_2_ = *n*-octyl).

The synthetic routes of the monomers and copolymers are shown in Scheme 1. The monomers M2 and M4 were synthesized by reported methods [31,32,34]; M1 was synthesized via the Horner-Wadsworth-Emmons reaction of diisopropyl ((5,5″-dibromo-[2,2′:5′,2″-terthiophen]-3′-yl)methyl)phosphonate 1 with 4,4″-bis(2-ethylhexyl)-[2,2′:5′,2″-terthiophene]-3′-carbaldehyde 2. The monomer M3 was synthesized from the bromination of 5-octyl-1,3-di(thiophen-2-yl)-4*H*-thieno[3,4-*c*]pyrrole-4,6(5*H*)-dione 4, which is the microwave-assisted Stille cross-coupling reaction product of 1,3-dichloro-5-octyl-4*H*-thieno[3,4-*c*]pyrrole-4,6(5*H*)-dione **3** and tributyl(thiophen-2-yl)stannane.

These three copolymers PTTVBDT, PTTVBDT-TPD, and PTTVBDT-DPP were performed by Stille coupling polymerization in chlorobenzene (CB) at 110 °C using Pd_2_dba_3_ and P(*o*-tol)_3_ as the catalyst and ligand, respectively. In the preparation of PTTVBDT-TPD and PTTVBDT-DPP, the feed molar ratio of M1 to the corresponding donor and the acceptor monomer (TPD or DPP units) in the reaction is 4:5:1. In other words, 2,6-bis(trimethyltin)-4,8-bis(2-ethylhexyloxy)benzo[1,2-b:4,5-b′]dithiophene (M2) was copolymerized with 80 mol % of the dibromo donors (M1) and 20 mol % of the dibromo acceptor, M3 and M4, to generate the corresponding copolymer PTTVBDT-TPD and PTTVBDT-DPP, respectively. The raw products were precipitated into methanol and collected through a Soxhlet thimble by filtration, which was then subjected to repeated Soxhlet extraction with methanol, acetone, hexane, and methylene chloride to remove the small molecules and oligomers, and finally with chloroform to collect the target compounds. Due to the presence of 2-ethylhexyl or *n*-octyl groups in the monomer units, all the copolymers are soluble in common organic solvents such as chloroform, THF, and chlorobenzene. More detailed synthetic procedures and characterization data of monomers and polymers are described in the Experimental Section and Supplementary Materials (Figures S1–S18) [35,36,37].

The number-average molecular weights (*M*_n_) of the resulting polymers, PTTVBDT, PTTVBDT-TPD, and PTTVBDT-DPP, were 47.1, 110.3, and 120.8 kDa, with a polydispersity index (PDI) of 2.29, 2.81, and 2.49, respectively, as determined by gel-permeation chromatography (GPC) using THF as the eluent at 40 °C (shown in Table 1). Most PTTVBDT was obtained by Soxhlet extractions in dichloromethane; in contrast, trace oligomers of PTTVBDT-TPD and PTTVBDT-DPP were removed by Soxhlet extractions in dichloromethane and most products were obtained in chloroform, which might probably cause the difference in molecular weight. In addition, the actual compositions of the copolymers were verified by elemental analyses: the composition (*m*/*n*) for PTTVBDT-TPD and PTTVBDT-DPP was 5.9 and 7.0, respectively.

As shown in Figure S19a, thermogravimetric analysis (TGA) curves of polymers, which were recorded under N_2_ atmosphere at a heating rate of 10 °C/min, show that all polymers had good thermal stability with degradation temperatures (*T*_d_) at 5% weight loss well beyond 350 °C (the measured data shown in Table 1). However, differential scanning calorimetry (DSC) analysis, shown in Figure S19b, of all the polymers reveals there are no apparent thermal transitions, such as T_g_ and T_m_, before 240 °C that may be due to the rigid structure of the polymer which is quite common in low-bandgap conjugated polymers [38].

**Figure 3:**
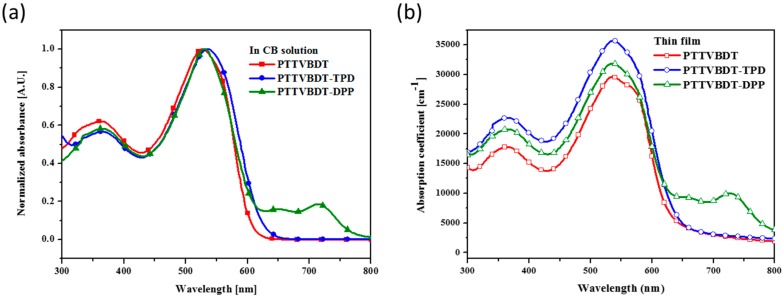
UV–vis absorption spectra of PTTVBDT, PTTVBDT-TPD, and PTTVBDT-DPP (**a**) in dilute chlorobenzene solution; (**b**) thin film (spin-coated from chlorobenzene solution and annealed for 10 min at 130 °C).

The optical properties of the polymers were examined by ultraviolet–visible (UV–vis) absorption spectroscopy. The UV–vis spectra of the polymers in dilute chlorobenzene solution and in spin-coated thin films on glass substrates are shown in Figure 3 and the corresponding absorption data of the polymers are summarized in Table 2. In the solution, the copolymer PTTVBDT exhibits two characteristic bands in the absorption spectra: the lower energy band with absorption peaked at ca. 526 nm corresponded to the π–π* transition of the conjugated backbone, while the shorter wavelength absorption band peaked at ca. 362 nm originated from the terthiophene units. Compared to the absorption spectra in the solution, the spectra of PTTVBDT in thin film which the wavelength of maximum absorption (*λ*_max_) and absorption edge (*λ*_edge_) were 536 and 635 nm, respectively, displaying broadened and red-shifted absorption attributed to the intermolecular interaction and aggregation in the solid state. Furthermore, by replacing TTV unit with electron acceptor TPD or DPP unit, a slightly broad and red-shift spectra of PTTVBDT-TPD with absorption edge measured at 647 nm was observed due to addition of the intramolecular charge transfer (ICT) interaction between the TPD and BDT. However, the absorption bands of the TPD containing polymers PTTVBDT-TPD are slightly broadened compared to PTTVBDT but are much narrower than for DPP-containing polymers PTTVBDT-DPP because TPD is a weaker acceptor than DPP [39,40]. In addition, Mario Leclerc et al. have reported a series of PBDTBTTPD derivatives which display absorption bands from around 300 to 650 nm with λ_max_ at ca. 516–553 nm, implying that the presence of electron-rich spacer in TPD-based conjugated polymers leads to a weaker intramolecular charge transfer [41]. Therefore, PTTVBDT-TPD displays slighter redshift and broadening of the absorption bands than PTTVBDT. On the other hand, PTTVBDT-DPP possesses the broadest absorption spectra and displays a significant absorption band around 600–800 nm which is derived from ICT interaction between the DPP and BDT, implying that DPP-BDT have stronger ICT interaction than TPD-BDT. As similar to PTTVBDT, the absorption spectra of PTTVBDT-TPD and PTTVBDT-DPP also displayed broadened and red-shifted absorption in the solid state than solution state.

In addition, the absorption edges of PTTVBDT, PTTVBDT-TPD and PTTVBDT-DPP thin films were located at 635, 647 and 791 nm, corresponding to optical band gaps (E_g, opt._) of 1.95, 1.91 and 1.73 eV, respectively. It is worthy to mention that the two ternary copolymers PTTVBDT-TPD and PTTVBDT-DPP exhibit higher absorption coefficients than PTTVBDT in thin film due to the introduction of TPD and DPP units and higher molecular weight. These observations suggest that they can have great potential for more effective light-harvesting and achieve a higher short-circuit current in PSCs.

**Figure 4:**
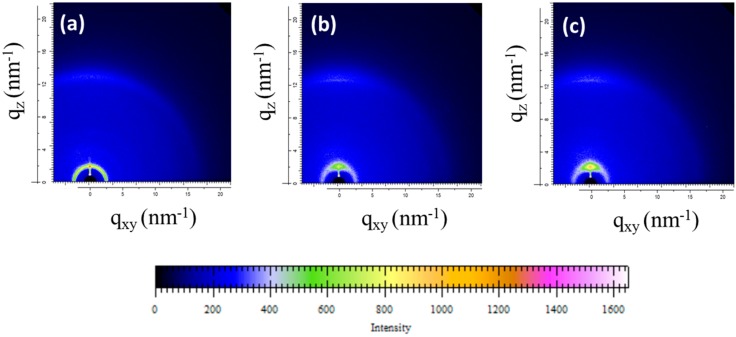
2D-GIXRD images of thin films of (**a**) PTTVBDT; (**b**) PTTVBDT-TPD; (**c**) PTTVBDT-DPP which were prepared by drop-casting and then thermal annealing at 120 °C, 20 min.

The orientation of conjugated polymers has a direct effect on their charge transport, leading to performance of photovoltaic and other organic electronic applications. It is well-known that investigating the relationship between polymer orientation and chemical structure is a critical and fundamental issue by molecular design. Herein, the two-dimensional grazing incidence X-ray diffraction (2D-GIXRD) technique was used to understand the influence of the introduction of acceptors on crystallinity, π–π stacking, and polymer-packing orientation relative to the substrate in BDT-based PTTVs’ system. The neat polymer films were prepared by drop-casting on SiO_2_ wafers and then thermal annealing at 120 °C, 20 min. The 2D-GIXRD experimental results are shown in Figure 4. The 2D-GIXRD pattern of PTTVBDT shows an isotropic ring of intensity at *q* ≈ 0.28 Å^−1^, which means it possesses randomly oriented lamellar stacks in thin film. In other words, due to absence of a preferential lamellar stacking orientation relative to the substrate, the face-on and edge-on orientation both appear to produce a ring of uniform intensity in 2D-GIXRD pattern. In contrast, the 2D-GIXRD patterns of PTTVBDT-TPD and PTTVBDT-DPP show a partial arc at *q*_z_ ≈ 0.30 and 0.31 Å^−1^, respectively, implying that these two terpolymers have not only a preferential orientation which may be beneficial to charge transport in PSCs but also closer lamellar packing behaviors.

The 2D diffraction patterns are further converted to the out-of-plane X-ray diffraction profile by fit-2D program, as shown in Figure S20. PTTVBDT, PTTVBDT-TPD and PTTVBDT-DPP exhibit (*d*_100_) diffraction peaks in the 2θ range of 2°–5°. For a further precise comparison, PTTVBDT-TPD and PTTVBDT-DPP thin film possess weaker intensity and broader diffraction peaks with FWHM (full-width at half-maximum of the X-ray diffraction peak) = 1.10° and 1.13°, respectively, corresponding to average crystallite sizes of 6.2 and 6.1 nm, respectively; meanwhile, the lamellar *d*-spacing (*d*_100_) for PTTVBDT-TPD is also calculated as 20.9 Å and for PTTVBDT-DPP as 20.6 Å, indicating PTTVBDT-TPD and PTTVBDT-DPP have reduced lamellar *d*-spacing, lower degree of crystallinities and smaller average crystalline grains in (100) plane than PTTVBDT (a lamellar *d*-spacing of 22.4 Å with a grain size of 12.7 nm). The different size, shape, and position of alkyl chains in the chemical structures of PTTVBDT-derivatives might probably cause the difference in the lamellar *d*-spacing. Regardless, these results reveal that the PTTVBDT-based polymer exhibit ordered orientations which is a critical property in conjugated polymers for application in electronic devices, especially PTTVBDT-TPD and PTTVBDT-DPP, displaying a preferred molecular orientation. The hole mobility of these polymers was measured by space charge limit current (SCLC) method, as listed in Table S1. PTTVBDT-TPD and PTTVBDT-DPP demonstrated hole mobilities of 6.04 × 10^−4^ and 4.63 × 10^−4^ cm^2^·V^−1^·s^−1^, respectively, which is about 10 times higher than that (5.88 × 10^−5^ cm^2^·V^−1^·s^−1^) of PTTVBDT, implying the preferred molecular orientation in PTTVBDT-TPD and PTTVBDT-DPP is beneficial to the direction of charge transport in the SCLC method.

**Figure 5:**
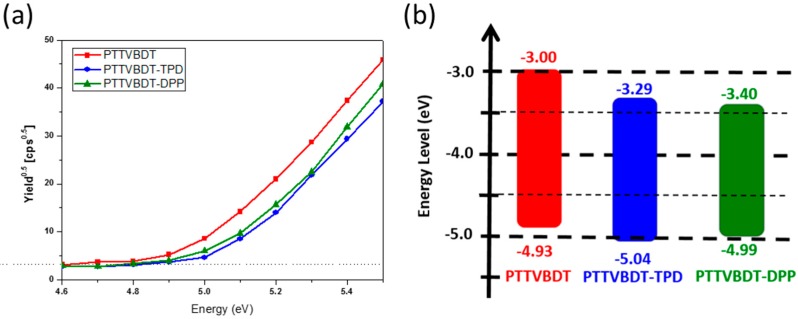
(**a**) Work function of polymer thin films determined by photoelectron emission; (**b**) Energy diagram of PTTVBDT, PTTVBDT-TPD, and PTTVBDT-DPP.

The highest occupied molecular orbital (HOMO) energies were estimated from the ionization potentials of spin-coated films determined by UV photoelectron spectroscopy in air using an AC2 photoelectron spectrometer (Riken Keiki Co.) [42]. The photoemission threshold energy, also called the work function, was determined from the crossing point of the background and the yield lines. Figure 5a plots the square root of the yield (cps^0.5^) as a function of the photon energy. Therefore, the work functions of PTTVBDT, PTTVBDT-TPD, and PTTVBDT-DPP were found to be −4.93 eV, −5.04 eV, and −4.99 eV, respectively. For comparison, the HOMO value of P3HT is −4.69 eV under identical conditions. These results reveal that our series of conjugated copolymers have lower HOMO energies than rr-P3HT; this will be beneficial for the fabrication of PSCs with high *V*_oc_ values. Furthermore, the lowest unoccupied molecular orbital (LUMO) energies were calculated using the optical band gap (E_g, opt._) and the HOMO energies according to the following equation: LUMO = HOMO + E_g, opt._ (eV). Therefore, the energy-level diagrams of polymers derived from AC2 photoelectron spectroscopy and UV–vis absorption data are shown in Figure 5b.

By comparing with the HOMO of PTTVBDT, the HOMO energies of PTTVBDT-TPD and PTTVBDT-DPP are 0.11 and 0.06 eV, respectively, deeper due to the TTV unit being replaced to the stronger acceptor unit which reduces electron-donating ability. On the other hand, the LUMO level is primarily affected by the electron-deficient units in general donor-acceptor polymers. Hence, the order of the LUMO levels is PTTVBDT-DPP (−3.40 eV) < PTTVBDT-TPD (−3.29 eV) < PTTVBDT (−3.00 eV), which not only is consistent with the acceptor strength of the introduced units but also agrees with the results of UV–vis absorption spectra. Moreover, the differences (>0.5 eV) between the LUMO energies of the polymers and PC_61_BM (ca. 4.20 eV) should allow for efficient charge carrier generation to occur in the devices by overcoming Coulombic binding energy of the exciton, since it is normally accepted that the LUMO energies of the donor should be at least 0.3 eV higher than that of the acceptor in BHJ solar cells [9,43,44].

**Figure 6:**
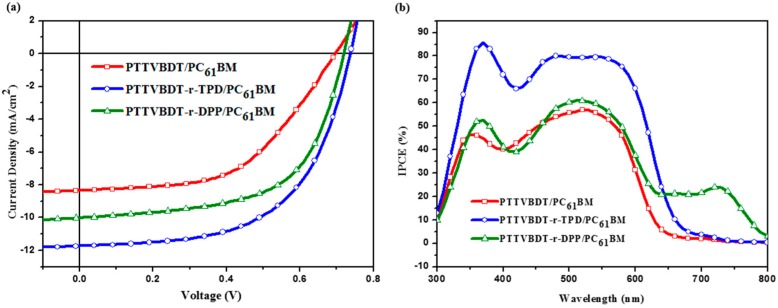
(**a**) *J–V* curves (**b**) IPCE spectra of optimized polymer/PC_61_BM devices under the illumination of AM 1.5G, 100 mW·cm^−2^.

To compare the photovoltaic properties of copolymers, the bulk heterojunction PSCs were fabricated with an conventional device configuration of ITO/PEDOT:PSS/polymer:PC_61_BM/Ca/Al. The current density–voltage (*J–V*) curves of the PSC devices under AM 1.5G illumination with an intensity of 100 mW·cm^−2^ are plotted in Figure 6a and the relevant average photovoltaic characteristics, including open-circuit voltage (*V*_oc_), short-circuit current (*J*_sc_), fill factor (*FF*), power conversion efficiency (PCE), series resistance (*R*_s_), and shunt resistance (*R*_sh_), are listed in Table 3. The optimum PCEs (%) for each PSCs are 3.10% (PTTVBDT/PC_61_BM 5:4 *w*/*w*), 5.01% (PTTVBDT-TPD/PC_61_BM 1:1 *w*/*w*), and 4.39% (PTTVBDT-DPP/PC_61_BM 5:4 *w*/*w*).

Since the *V*_oc_ value of PSCs is primarily proportional to the difference between the HOMO energy level of the donor polymer and the LUMO energy level of the fullerene acceptor, these devices prepared from the blends of PTTVBDT, PTTVBDT-TPD, and PTTVBDT-DPP with PC_61_BM exhibited average *V*_oc_ of 703, 740, and 723 mV, respectively, are in good agreement with the order of the HOMO energy levels of these polymers. Additionally, the *J*_sc_ of the devices based on blends of PTTVBDT, PTTVBDT-TPD, and PTTVBDT-DPP with PC_61_BM were 8.10, 11.65, and 9.96 mA·cm^−2^, respectively.

In order to investigate the improved *J*_sc_, the monochromatic incident photon-to-electron conversion efficiency (IPCE) spectra of the optimized polymer/PC_61_BM devices were measured under AM 1.5G illumination with an intensity of 100 mW·cm^−2^ and shown in Figure 6b. It is worth noting that due to the high efficiency of the photon-to-electron conversion for PTTVBDT-TPD/PC_61_BM (>60% over the range 340–600 nm and peaking at ca. 85% at 370 nm), they not only possess the highest *J*_sc_ in all the solar cell devices but are potential materials for photo-detector devices. In addition, the IPCE plot of PTTVBDT-DPP/PC_61_BM displays the broadest IPCE region extended to 800 nm, indicating that the incorporation of the strongest electron-withdrawing DPP units into the polymer backbone effectively improves the *J*_sc_ value in solar cell devices by enhancing absorption ability in comparison with PTTVBDT/PC_61_BM.

For photovoltaic devices, the charge mobility is a crucial factor for achieving highly efficient devices. Herein, the space-charge–limited current (SCLC) method with the device structures of ITO/PEDOT:PSS/blend film/Au for the hole mobility and ITO/SAM-NH_2_/blend film/Ca/Al for the electron mobility was applied to verify the hole and electron mobility of the blend films. As listed in Table S1, the hole/electron mobilities of PTTVBDT-TPD, and PTTVBDT-DPP blend films were determined to be 5.70 × 10^−4^/8.37 × 10^−4^ and 4.23 × 10^−4^/5.69 × 10^−4^ cm^2^·V^−1^·s^−1^, respectively, which are one order higher than PTTVBDT blend film of 2.01 × 10^−5^/3.65 × 10^−5^ cm^2^·V^−1^·s^−1^. This result corresponds to improved series resistance and fill factor for devices based on PTTVBDT-TPD and PTTVBDT-DPP, in comparison with PTTVBDT. Furthermore, the balanced charge transport of PTTVBDT-DPP blend film is closer to 1 than the others, explaining why it exhibits the highest FF and the lowest *R*_s_. Atomic force microscopy (AFM) was applied to verify the morphologies of the spin-coated films of polymers/PC_61_BM on top of ITO/PEDOT:PSS, which were prepared by using the same procedure and parameters for preparing the photoactive layers. As the phase images of the blend films were shown in Figure S21, the polymers/PC_61_BM blend films display very similar morphological properties. The root-mean-square (RMS) surface roughness of PTTVBDT blend films appears to be larger than that of the others, corresponding to the highest *R*_s_ due to the increased interfacial contact resistance.

## 4. Conclusions

In summary, we have successfully synthesized and characterized a novel p-type conjugated copolymer, PTTVBDT; cooperating BDT and TTV units via Stille polymerization. PTTVBDT shows a low-lying HOMO energy level and ordered molecular-packing behavior. Furthermore, by introducing different electron-withdrawing groups, two terpolymers, PTTVBDT-TPD and PTTVBDT-DPP; display stronger absorption ability, lower-lying HOMO energy level, and preferred molecular orientation, indicating the absorption ability, bandgaps, and molecular energy levels of the polymers can be further tuned by replacing TTV-monomer units with different acceptors. The polymer solar cells based on the blends of PTTVBDT, PTTVBDT-TPD, and PTTVBDT-DPP with PC_61_BM exhibited the best power conversion efficiencies of 3.10%, 5.01%, and 4.39%, respectively. This work demonstrates an effective strategy combining a two-dimensional molecular structure with random copolymerization strikes producing conjugated polymers to achieve highly efficient organic photovoltaic devices.

## Figures and Tables

**Table 1:** Polymerization results and thermal properties of copolymers.

Polymer	*M*_w_^a^ (kDa)	*M*_n_^a^ (kDa)	PDI ^a^	*m*/*n* ^b^	*T*_d_^c^ (°C)
PTTVBDT	124.6	47.1	2.64	-	357
PTTVBDT-TPD	309.4	110.3	2.81	5.9	358
PTTVBDT-DPP	300.6	120.8	2.49	7.0	368

^a^ *M*_w_, *M*_n_, and PDI of the polymers were estimated by GPC using polystyrene as standards in THF; ^b^ *m*/*n* ratio was calculated by elemental analyses; ^c^ 5% weight-loss temperature measured by TGA under N_2_ atmosphere.

**Table 2:** Optical and electrochemical properties of the polymers.

Polymer	Solution, λ_max_ (nm) ^a^	Film, λ_max_ (nm) ^b^	λ_edge_ (nm) ^b^	E_g, opt._ (eV) ^c^	E_HOMO,AC2_ (eV) ^d^	E_LUMO_ (eV) ^e^
PTTVBDT	526	536	635	1.95	−4.93	−2.98
PTTVBDT-TPD	534	537	647	1.91	−5.04	−3.13
PTTVBDT-DPP	530	536	791	1.56	−4.99	−3.43

^a^ Measured in dilute CB solution (<10^−3^ g/L); ^b^ Samples were prepared by spin-coating from CB solution (10 g/L) on glass substrate and then thermal annealed at 120 °C, 20 min; ^c^ Estimated from the onset wavelength of the thin-film absorption spectra: E_g, opt._ = 1240/λ_onset_; ^d^ HOMO energy levels were evaluated by ultraviolet photoelectron spectroscopy (AC2 equipment) from UV–vis thin-film samples; ^e^ Calculated according to the equation: LUMO = HOMO + E_g, opt._ (eV).

**Table 3:** Photovoltaic properties of polymer solar cell devices based on the polymers, PTTVBDT, PTTVBDT-TPD, and PTTVBDT-DPP, with optimized ratios of PC_61_BM under the illumination of AM 1.5G, 100 mW·cm^−2^.

Polymer/fullerene	*V*_oc_ (mV)	*J*_sc_ (mA/cm^2^)	FF (%)	PCE (%)		*R*_sh_ (kΩ·cm^2^)	*R*_s_ (Ω·cm^2^)
				Avg.	Best		
PTTVBDT/PC_61_BM ^a^	703 ± 13	8.10 ± 0.06	52.24 ± 1.08	2.98	3.10	0.89	29.17
PTTVBDT-TPD/PC_61_BM ^b^	740 ± 0	11.65 ± 0.08	57.63 ± 0.03	4.97	5.01	1.05	9.78
PTTVBDT-DPP/PC_61_BM ^c^	723 ± 4	9.96 ± 0.21	59.5 ± 0.4	4.29	4.39	0.84	8.56

^a^ Blend ratio 5:4, in CB solvent, annealed at 90 °C for 10 min; ^b^ blend ratio 1:1, in CB solvent, annealed at 100 °C for 10 min; ^c^ blend ratio 5:4, in *o*-DCB solvent, annealed at 100 °C for 10 min.

## Supplementary Materials

The following are available online at www.mdpi.com/2073-4360/8/11/382/s1. Figure S1: ^1^H-NMR spectrum of Compound S2; Figure S2: ^13^C-NMR spectrum of Compound S2; Figure S3: ^1^H-NMR spectrum of Compound S3; Figure S4: ^13^C-NMR spectrum of Compound S3; Figure S5: ^1^H-NMR spectrum of Compound S4; Figure S6: ^13^C-NMR spectrum of Compound S4; Figure S7: ^1^H-NMR spectrum of Compound 1; Figure S8: ^13^C-NMR spectrum of Compound 1; Figure S9: ^13^C-NMR spectrum of Compound S6; Figure S10: ^13^C-NMR spectrum of Compound S7; Figure S11: ^1^H-NMR spectrum of Compound 3; Figure S12: ^13^C-NMR spectrum of Compound 3; Figure S13: ^1^H-NMR spectrum of Compound 4; Figure S14: ^13^C-NMR spectrum of Compound 4; Figure S15: ^1^H-NMR spectrum of Compound M1; Figure S16: ^13^C-NMR spectrum of Compound M1; Figure S17: ^1^H-NMR spectrum of Compound M3; Figure S18: ^13^C-NMR spectrum of Compound M3; Figure S19: (a) TGA and (b) DSC second heating profiles of PTTVBDT, PTTVBDT-TPD, and PTTVBDT-DPP with a heating rate of 10 °C/min under N_2_ atmosphere and a cooling rate of 10 °C/min; Figure S20: 1D-GIXRD out-of-plane of PTTVBDT, PTTVBDT-TPD, and PTTVBDT-DPP films which were prepared by drop-casting and then thermal annealing at 120 °C, 20 mins; Figure S21: AFM (a–c) topography images and (d–f) phase images of spin-coated films of polymer/PC_61_BM blends. The scan sizes for all images are 2 μm × 2 μm. Table S1: Mobility of PTTVBDT, PTTVBDT-TPD, and PTTVBDT-DPP with/without PC_61_BM by the SCLC method.

## References

[1] Facchetti A. (2007). Semiconductors for organic transistors. Mater. Today.

[2] Yuan J., Huang X., Zhang F., Lu J., Zhai Z., Di C., Jiang Z., Ma W. (2012). Design of benzodithiophene-diketopyrrolopyrrole based donor-acceptor copolymers for efficient organic field effect transistors and polymer solar cells. J. Mater. Chem..

[3] Qian G., Qi J., Wang Z.Y. (2012). Synthesis and study of low-bandgap polymers containing the diazapentalene and diketopyrrolopyrrole chromophores for potential use in solar cells and near-infrared photodetectors. J. Mater. Chem..

[4] Grimsdale A.C., Leok Chan K., Martin R.E., Jokisz P.G., Holmes A.B. (2009). Synthesis of light-emitting conjugated polymers for applications in electroluminescent devices. Chem. Rev..

[5] AlSalhi M.S., Alam J., Dass L.A., Raja M. (2011). Recent advances in conjugated polymers for light emitting devices. Int. J. Mol. Sci..

[6] Usta H., Sheets W.C., Denti M., Generali G., Capelli R., Lu S., Yu X., Muccini M., Facchetti A. (2014). Perfluoroalkyl-functionalized thiazole–thiophene oligomers as n-channel semiconductors in organic field-effect and light-emitting transistors. Chem. Mater..

[7] Li G., Zhu R., Yang Y. (2012). Polymer solar cells. Nat. Photonics.

[8] Zhang S., Ye L., Hou J. (2016). Breaking the 10% efficiency barrier in organic photovoltaics: Morphology and device optimization of well-known pbdttt polymers. Adv. Energy Mater..

[9] Son H.J., Carsten B., Jung I.H., Yu L. (2012). Overcoming efficiency challenges in organic solar cells: Rational development of conjugated polymers. Energy Environ. Sci..

[10] Li Y. (2012). Molecular design of photovoltaic materials for polymer solar cells: Toward suitable electronic energy levels and broad absorption. Acc. Chem. Res..

[11] Boudreault P.-L.T., Najari A., Leclerc M. (2010). Processable low-bandgap polymers for photovoltaic applications. Chem. Mater..

[12] Kuo C.-Y., Nie W., Tsai H., Yen H.-J., Mohite A.D., Gupta G., Dattelbaum A.M., William D.J., Cha K.C., Yang Y. (2014). Structural design of benzo[1,2-b:4,5-b′]dithiophene-based 2d conjugated polymers with bithienyl and terthienyl substituents toward photovoltaic applications. Macromolecules.

[13] Liao S.-H., Jhuo H.-J., Cheng Y.-S., Chen S.-A. (2013). Fullerene derivative-doped zinc oxide nanofilm as the cathode of inverted polymer solar cells with low-bandgap polymer (PTB7-Th) for high performance. Adv. Mater..

[14] Wang N., Chen W., Shen W., Duan L., Qiu M., Wang J., Yang C., Du Z., Yang R. (2016). Novel donor-acceptor polymers containing o-fluoro-p-alkoxyphenyl-substituted benzo[1,2-b:4,5-b′]dithiophene units for polymer solar cells with power conversion efficiency exceeding 9%. J. Mater. Chem. A.

[15] Huang J., Carpenter J.H., Li C.-Z., Yu J.-S., Ade H., Jen A.K.Y. (2016). Highly efficient organic solar cells with improved vertical donor–acceptor compositional gradient via an inverted off-center spinning method. Adv. Mater..

[16] Hou J., Yang C., He C., Li Y. (2006). Poly[3-(5-octyl-thienylene-vinyl)-thiophene]: A side-chain conjugated polymer with very broad absorption band. Chem. Commun..

[17] Hou J., Tan Z.A., Yan Y., He Y., Yang C., Li Y. (2006). Synthesis and photovoltaic properties of two-dimensional conjugated polythiophenes with bi(thienylenevinylene) side chains. J. Am. Chem. Soc..

[18] Zou Y., Wu W., Sang G., Yang Y., Liu Y., Li Y. (2007). Polythiophene derivative with phenothiazine−vinylene conjugated side chain:  Synthesis and its application in field-effect transistors. Macromolecules.

[19] Zou Y., Sang G., Wu W., Liu Y., Li Y. (2009). A polythiophene derivative with octyloxyl triphenylamine-vinylene conjugated side chain: Synthesis and its applications in field-effect transistor and polymer solar cell. Synth. Metals.

[20] Hsiow C.-Y., Raja R., Wang C.-Y., Lin Y.-H., Yang Y.-W., Hsieh Y.-J., Rwei S.-P., Chiu W.-Y., Huang C.-I., Wang L. (2014). Impact of constitution of the terthiophene-vinylene conjugated side chain on the optical and photovoltaic properties of two-dimensional polythiophenes. Phys. Chem. Chem. Phys..

[21] Duan R., Ye L., Guo X., Huang Y., Wang P., Zhang S., Zhang J., Huo L., Hou J. (2012). Application of two-dimensional conjugated benzo[1,2-b:4,5-b′]dithiophene in quinoxaline-based photovoltaic polymers. Macromolecules.

[22] Gu Z., Shen P., Tsang S.-W., Tao Y., Zhao B., Tang P., Nie Y., Fang Y., Tan S. (2011). Development of a new benzo(1,2-b:4,5-b′)dithiophene-based copolymer with conjugated dithienylbenzothiadiazole-vinylene side chains for efficient solar cells. Chem. Commun..

[23] Wang C., Zhao B., Cao Z., Shen P., Tan Z., Li X., Tan S. (2013). Enhanced power conversion efficiencies in bulk heterojunction solar cells based on conjugated polymer with isoindigo side chain. Chem. Commun..

[24] Shen P., Bin H., Xiao L., Li Y. (2013). Enhancing photovoltaic performance of copolymers containing thiophene unit with D–A conjugated side chain by rational molecular design. Macromolecules.

[25] Kuo C.-Y., Huang Y.-C., Hsiow C.-Y., Yang Y.-W., Huang C.-I., Rwei S.-P., Wang H.-L., Wang L. (2013). Effect of side-chain architecture on the optical and crystalline properties of two-dimensional polythiophenes. Macromolecules.

[26] Hsiow C.-Y., Lin Y.-H., Raja R., Rwei S.-P., Chiu W.-Y., Dai C.-A., Wang L. (2016). Modified structure of two-dimensional polythiophene derivatives by incorporating electron-deficient units into terthiophene-vinylene conjugated side chains and the polymer backbone: Synthesis, optoelectronic and self-assembly properties, and photovoltaic application. RSC Adv..

[27] Cabanetos C., El Labban A., Bartelt J.A., Douglas J.D., Mateker W.R., Fréchet J.M.J., McGehee M.D., Beaujuge P.M. (2013). Linear side chains in benzo[1,2-b:4,5-b′]dithiophene–thieno[3,4-c]pyrrole-4,6-dione polymers direct self-assembly and solar cell performance. J. Am. Chem. Soc..

[28] Kim K.-H., Park S., Yu H., Kang H., Song I., Oh J.H., Kim B.J. (2014). Determining optimal crystallinity of diketopyrrolopyrrole-based terpolymers for highly efficient polymer solar cells and transistors. Chem. Mater..

[29] Shi H., Fu W., Shi M., Ling J., Chen H. (2015). A solution-processable bipolar diketopyrrolopyrrole molecule used as both electron donor and acceptor for efficient organic solar cells. J. Mater. Chem. A.

[30] Cheon Y.R., Kim Y.J., Ha J.-J., Kim M.-J., Park C.E., Kim Y.-H. (2014). Tpd-based copolymers with strong interchain aggregation and high hole mobility for efficient bulk heterojunction solar cells. Macromolecules.

[31] Liang Y., Feng D., Wu Y., Tsai S.-T., Li G., Ray C., Yu L. (2009). Highly efficient solar cell polymers developed via fine-tuning of structural and electronic properties. J. Am. Chem. Soc..

[32] Huo L., Hou J., Chen H.-Y., Zhang S., Jiang Y., Chen T.L., Yang Y. (2009). Bandgap and molecular level control of the low-bandgap polymers based on 3,6-dithiophen-2-yl-2,5-dihydropyrrolo[3,4-c]pyrrole-1,4-dione toward highly efficient polymer solar cells. Macromolecules.

[33] Robinson D.M., Go Y.B., Greenblatt M., Dismukes G.C. (2010). Water oxidation by λ-mno2: Catalysis by the cubical Mn_4_O_4_ subcluster obtained by delithiation of spinel LiMn_2_O_4_. J. Am. Chem. Soc..

[34] Liang Y., Wu Y., Feng D., Tsai S.-T., Son H.-J., Li G., Yu L. (2009). Development of new semiconducting polymers for high performance solar cells. J. Am. Chem. Soc..

[35] Tan H., Deng X., Yu J., Zhao B., Wang Y., Liu Y., Zhu W., Wu H., Cao Y. (2012). A novel benzo[1,2-b:4,5-b′]dithiophene-based conjugated polymer with a pendant diketopyrrolopyrrole unit for high-performance solar cells. Macromolecules.

[36] Ayres B.E., Longworth S.W., McOmie J.F.W. (1975). Synthesis of derivatives of cyclobuteno[c]thiophen and attempts to synthesise thiophen analogues of biphenylene. Tetrahedron.

[37] Yuan M.-C., Chiu M.-Y., Liu S.-P., Chen C.-M., Wei K.-H. (2010). A thieno[3,4-c]pyrrole-4,6-dione-based donor−acceptor polymer exhibiting high crystallinity for photovoltaic applications. Macromolecules.

[38] Patil A.V., Lee W.-H., Lee E., Kim K., Kang I.-N., Lee S.-H. (2011). Synthesis and photovoltaic properties of a low-band-gap copolymer of dithieno[3,2-b:2′,3′-d]thiophene and dithienylquinoxaline. Macromolecules.

[39] Khlyabich P.P., Burkhart B., Rudenko A.E., Thompson B.C. (2013). Optimization and simplification of polymer–fullerene solar cells through polymer and active layer design. Polymer.

[40] Zhang Z.-G., Wang J. (2012). Structures and properties of conjugated donor-acceptor copolymers for solar cell applications. J. Mater. Chem..

[41] Najari A., Beaupré S., Berrouard P., Zou Y., Pouliot J.-R., Lepage-Pérusse C., Leclerc M. (2011). Synthesis and characterization of new thieno[3,4-c]pyrrole-4,6-dione derivatives for photovoltaic applications. Adv. Funct. Mater..

[42] Chang Y.-M., Wang L., Su W.-F. (2008). Polymer solar cells with poly(3,4-ethylenedioxythiophene) as transparent anode. Org. Electron..

[43] Liu Y., Chen C.-C., Hong Z., Gao J., Yang Y., Zhou H., Dou L., Li G., Yang Y. (2013). Solution-processed small-molecule solar cells: Breaking the 10% power conversion efficiency. Sci. Rep..

[44] Cheng Y.-J., Yang S.-H., Hsu C.-S. (2009). Synthesis of conjugated polymers for organic solar cell applications. Chem. Rev..

