# Supplementary material for: Synthesis and Characterization of Two-Dimensional Conjugated Polymers Incorporating Electron-Deficient Moieties for Application in Organic Photovoltaics
Source: Polymers (Basel). 2016 Oct 27;8(11):382. doi: 10.3390/polym8110382 (PMC6432402; doi:10.3390/polym8110382)
Supplement: Supplementary file 1 [file polymers-08-00382-s001.pdf]

Supplementary Materials: Synthesis and Characterization of Two-Dimensional Conjugated Polymers Incorporating Electron-Deficient Moieties for Application in Organic Photovoltaics

Chuen-Yo Hsiow, Han-Ying Wang, Yu-Hsiang Lin, Rathinam Raja, Syang-Peng Rwei, Wen-Yen Chiu, Chi-An Dai and Leeyih Wang

1. Materials, Instrument, Device Fabrication and Characterization

1.1. Materials

All reagents were purchased from Acros, Aldrich, and Alfa Aesar and used without further purification unless otherwise noted. 2,6-Bis(trimethyltin)-4,8-bis(2-ethylhexyloxy)benzo[1,2-*b*:4,5-b′]dithiophene (M2) [31] and 3,6-bis(5-bromothiophen-2-yl)-2,5-bis(2-ethylhexyl)pyrrolo[3,4-*c*]pyrrole-1,4(2*H*,5*H*)-dione (M3) [32] were prepared via literature procedures.

1.2. Instrument and Characterization

^{1}H NMR and ^{13}C NMR spectra were recorded in chloroform-*d* ($CDCl_3$) solution at 400 MHz on Bruker DRX-400 spectrometer. All NMR spectra were calibrated by $CDCl_3$ where ^{1}H NMR chemical shifts of $CDCl_3$ is 7.23 ppm; in addition, ^{13}CNMR of $CDCl_3$ is 77.0 ppm. Microwave assisted Stille cross-coupling reactions were performed in Anton Paar Monowave 300 microwave reactor. Ultraviolet-visible absorption (UV-vis) absorption spectra in solution or solid thin film were recorded on a JASCO MD-2010 spectrometer. Gel permeation chromatography (GPC) was conducted at 40 °C using two Jordi DVB mixed-bed columns (250 mm × 10 mm; suitable for separating polymers with molecular weights from 1×10^2 to 1×10^7 g·mol^{-1}) using THF as the eluent at a flow rate of 1.0 mL/min on a JASCO instrument that was equipped with UV-vis and refractive index (RI) detectors connected in series. The work functions of materials were measured using an AC2 photoelectron spectrometer (Riken Keiki Co.). Atomic force microscopy (AFM) images were captured using taping model in Digital Instruments Nanoscope III. The AFM samples were prepared by spin-coating the blend solution on a ITO/PEDOT:PSS substrate.

1.3. Device Fabrication and Characterization

The polymer solar cells (PSCs) were fabricated with the structure of ITO/PEDOT:PSS/polymer: $PC_{61}BM$/Ca/Al by our previous work [20,26]. The ITO glasses were cleaned by a sequential ultrasonic treatment in detergent, deionized water, acetone, and isopropanol for 20 min. Then PEDOT:PSS was filtered through a 0.2-μm filter and spin-coated at 3500 rpm for 30 s on top of ITO electrode. Subsequently, the PEDOT:PSS film was baked at 140 °C for 10 min in the air, and then moved into a glovebox. The blend solution of $PC_{61}BM$ and synthesized polymers in a solvent was filtered with/without a 0.45-μm filter and spin-coated at 800 rpm for 30 s on top of the PEDOT:PSS layer. These devices were thermally annealed at various temperatures for 10 min, followed by capping with Ca (~20 nm) and then Al (~60 nm) in a thermal evaporator at a base pressure of ca. 10^{-6} Pa. The active area of the devices is 0.06 cm^2. The current density−voltage (J−V) measurements of the devices were conducted on a computer-controlled Keithley 2400 Source Measure Unit under AM 1.5 G simulated solar irradiation at 100 mW·cm^{-2}. The light incident intensity was calibrated by a mono-Si reference cell with a KG5 filter (PV Measurements, Inc.), which was pre-calibrated by the National Renewable Energy Laboratory. The SCLC measurements were carried out using a Keithley 2400 source meter under dark condition. The IPCE spectra were recorded under illumination by a xenon lamp and a monochromator (TRIAX 180, JOBIN YVON), and the light intensity was calibrated by using an OPHIR 2A-SH thermopile detector.

2. Synthesis

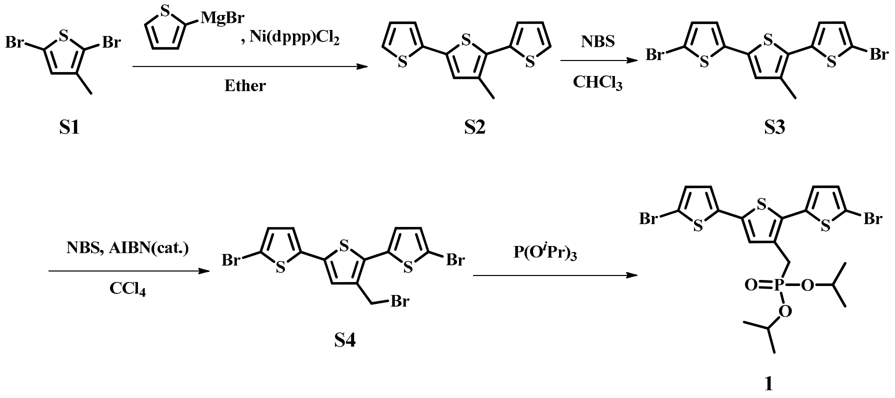

Scheme S1. Synthetic route of **1** (R_1 = 2-ethylhexyl).

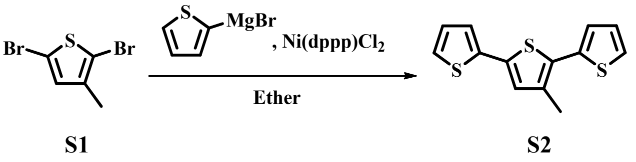

3′-methyl-2,2′:5′,2″-terthiophene (S2). Take magnesium turnings (9.0 g, 375.0 mmol) in a 500 mL double-neck bottle with condenser and then degassed via vacuum-nitrogen cycle by three times. After injecting 150 mL anhydrous ether under nitrogen, 2-bromothiophene (18.0 mL, 185.5 mmol) was added and then it refluxed 2 h. The mixture was cooled to room temperature and transferred to a solution containing 2,5-dibromo-3-methylthiophene S1 (9.0 g, 35.1 mmol), dichloro[1,3-bis (diphenylphosphino)propane]nickel (0.1 g, 0.18 mmol) and 150 mL anhydrous ether in ice bath. After stirred overnight at room temperature, the solution was added 150 mL saturated ammonium chloride aqueous and stirred 30 min. Extracted twice with 100 mL hexane and the organic phase was combined and dried with $MgSO_4$. After removal of the solvent, the residue was purified by column chromatography (eluent: hexane, R_f = 0.5), yielding a light yellow liquid S2 (6.3 g, 23.9 mmol, 68%). ^{1}H NMR (400 MHz, $CDCl_3$) δ 7.29 (dd, *J* = 5.12, 0.92 Hz, 1H), 7.20 (dd, *J* = 5.12, 0.92 Hz, 1H), 7.15–7.14 (m, 2H), 7.06 (dd, *J* = 5.12, 5.08 Hz, 1H), 7.00 (dd, *J* = 5.12, 5.08 Hz, 1H), 6.97 (s, 1H), 2.37 (s, 3H); ^{13}C NMR (100 MHz, $CDCl_3$) δ 137.12, 136.30, 134.65, 134.52, 130.02, 127.86, 127.83, 127.45, 125.39, 125.09, 124.34, 123.56, 15.47.

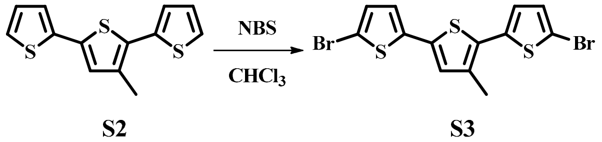

5,5″-dibromo-3′-methyl-2,2′:5′,2″-terthiophene (S3). A mixture of S2 (5.5 g, 20.9 mmol) and *N*-bromosuccinimide (8.2 g, 46.0 mmol) in 100 mL chloroform stirred overnight at room temperature. Then the solution was poured into water and extracted twice with 50 mL dichloromethane. The organic phase was combined and dried with $MgSO_4$. After removal of the solvent, the residue was purified by flash column chromatography (eluent: hexane/CH_2Cl_2 = 15/1, R_f = 0.6), yielding yellow solid S3 (3.5 g, 8.3 mmol, 39%). ^{1}H NMR (400 MHz, $CDCl_3$) δ 7.01 (d, *J* = 3.84 Hz, 2H), 6.96 (d, *J* = 3.88 Hz, 2H), 6.88–6.86 (m, 3H), 2.32 (s, 3H); ^{13}C NMR (100 MHz, $CDCl_3$) δ 138.32, 137.51, 135.11, 134.08, 130.70, 130.30, 129.47, 127.98, 125.72, 123.80, 111.96, 111.17, 15.35.

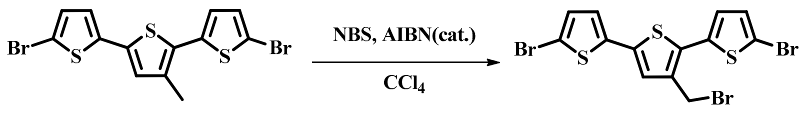

S3 S4

5,5″-dibromo-3′-(bromomethyl)-2,2′:5′,2″-terthiophene (S4). A mixture S3 (4.0 g, 9.5 mmol) and *N*-bromosuccinimide (1.69 g, 9.5 mmol) was dissolved in 15 mL carbon tetrachloride. 2,2′-Azobis(2-methylpropionitrile) (0.04 g, 0.24 mmol) was added as a radical initiator, and then the mixture was refluxed overnight. The solution was poured into water at room temperature and extracted twice with 30 mL dichloromethane. The organic phase was combined and dried with $MgSO_4$. After removal of the solvent, the residue was purified by column chromatography (eluent: hexane/CH_2Cl_2 = 15/1, R_f = 0.6), yielding brown solid S4 (2.3 g, 4.6 mmol, 48%). ^1H NMR (400 MHz, $CDCl_3$) δ 7.07 (sd, *J* = 3.88 Hz, 2H), 7.05 (d, *J* = 3.88 Hz, 1H), 6.98, (d, *J* = 3.88 Hz, 1H), 6.92 (d, *J* = 3.88 Hz, 1H), 4.51 (s, 2H); ^{13}C NMR (100 MHz, $CDCl_3$) δ 137.47, 135.77, 135.20, 134.86, 132.66, 130.87, 130.77, 127.31, 126.30, 124.42, 113.76, 111.98, 26.00.

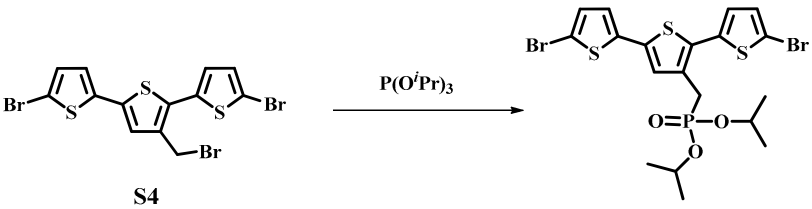

S4 1

Diisopropyl ((5,5″-dibromo-[2,2′:5′,2″-terthiophen]-3′-yl)methyl)phosphonate (1) [35]. S4 (2.3 g, 4.6 mmol), triisopropyl phosphite (3.4 mL, 13.7 mmol), and 50 mL toluene were put in a 100 mL two-neck round-bottom flask with condenser and then refluxed overnight. After cooling down to room temperature and removing most solvent, the crude product was purified by flash column chromatography (eluent: hexane:ethyl acetate = 1:1, R_f = 0.6) , yielding a light brown liquid **1** (2.3 g, 3.9 mmol, 85%). ^1H NMR (400 MHz, $CDCl_3$) δ 7.17 (d, *J* = 1.44 Hz, 1H), 7.09 (d, *J* = 3.84 Hz, 1H), 7.04 (d, *J* = 3.84 Hz, 1H), 6.97 (d, *J* = 3.84 Hz, 1H), 6.90 (d, *J* = 3.84 Hz, 1H), 4.64 (m, 2H), 3.17 (d, *J* = 21.36 Hz, 1H), 1.31 (d, *J* = 6.2 Hz, 1H), 1.21 (d, *J* = 6.2 Hz, 1H); ^{13}C NMR (100 MHz, $CDCl_3$) δ 137.92, 135.88, 134.99, 131.05, 130.93, 130.60, 129.35, 129.26, 127.54, 127.05, 123.91, 112.82, 111.38, 70.97, 70.90, 29.10, 27.67, 24.00, 24.97, 23.76, 23.71.

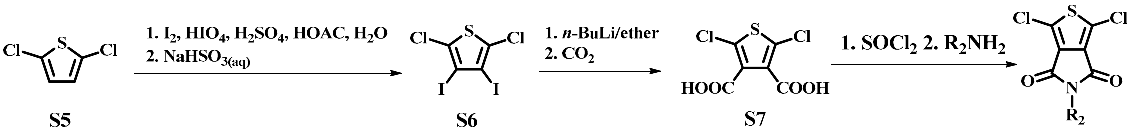

S5 S6 S7 3

Scheme S2. Synthetic route of monomer **3** (R_2 = *n*-octyl).

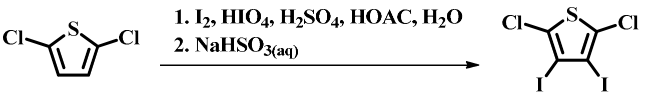

S5 S6

2,5-Dichloro-3,4-diiodothiophene (S6) [6]. The mixture of S5 (20.0 g, 130.7 mmol), iodine (27.6 g, 108.8 mmol), and periodic acid (12.0 g, 52.6 mmol) were dissolved in mixture consisting of 80 mL acetic acid and 6 mL deionized water in a 250 mL two-neck round-bottom flask with condenser. After 4 mL sulfuric acid was added dropwise, the reaction mixture was heated to 90 °C for 5 h. The dark

purple solution was cooled down to 60 °C and then $NaHSO_{3(aq)}$ (20.0 g $NaHSO_3$ in 100 mL deionized water) was added slowly and carefully, causing to a yellow solution. The mixture was stirred for another 1 h. After cooling down to ambient temperature, the mixture was filtered under vacuum on a Büchner funnel and washed sequentially with water. The pale yellow ball-like solid residue was dissolved in 40 mL DMSO and then reprecipitated in 400 mL 1.0 M $NaOH_{(aq)}$ at 0 °C. The mixture was stirred for another 5 min and then filtered immediately through a Büchner funnel. The solid is washed with 500 mL deionized water and then dried under vacuum for 8 h, yielding a white powder S6 (45.0 g, 111.2 mmol, 85%). ^{13}C NMR (100 MHz, $CDCl_3$) δ 128.53, 94.70.

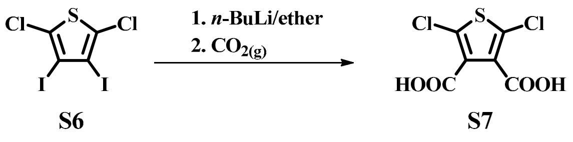

2,5-Dichlorothiophene-3,4-dicarboxylic acid (S7) [36]. *n*-BuLi (70.0 mL of 2.5 M solution in hexane, 175.0 mmol) was added dropwise to a solution of S6 (40.0 g, 98.8 mmol) in 120 mL anhydrous ether at -78°C, and then stir 2 h at the same temperature. Then it was quenched by dry $CO_{2(g)}$ for 2 h at −78 °C. After it was allowed warm slowly to ambient temperature, the mixture was poured into 300 mL 1.0 M $NaOH_{(aq)}$ and extracted twice with 100 mL ether. The aqueous phase was kept, acidified to pH 1–2 using a 2 M $HCl_{(aq)}$, and then extracted twice with 80 mL ether. The organic phase was combined and dried with $MgSO_4$. After removal of the solvent, the residue was dissolved in 40 mL ether and then reprecipitated in 400 mL hexane at 0 °C. The mixture was stirred for another 5 min and then filtered immediately under vacuum through a sintered-glass funnel. The solid is washed with 150 mL hexane and dried in oven-vacuum for 8 h at 60 °C, yielding a white powder S7 (17.5 g, 45.8 mmol, 46%). ^{13}C NMR (100 MHz, DMSO-d_6) δ 162.93, 132.83, 128.90.

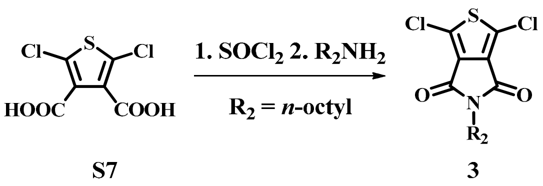

1,3-Dichloro-5-octyl-4*H*-thieno[3,4-*c*]pyrrole-4,6(5*H*)-dione (3) [37]. A solution of 2,5-dichlorothiophene-3,4-dicarboxylic acid S7 (10.0 g, 30.3 mmol) in 30 mL of thionyl chloride was heated to reflux for 5 h, then cooled to room temperature which displayed orange-brown color. After the removal of the volatiles under reduced pressure, the deep-brown residue solid was refluxed with *n*-octylamine (5.0 mL, 30.3 mmol) for 12 h. The solution was poured into water at room temperature and extracted twice with 40 mL dichloromethane. The organic phase was combined and dried with $MgSO_4$. After removal of the solvent, the residue was purified by column chromatography (eluent: hexane/ethyl acetate = 10/1), yielding white solid **3** (4.55 g, 13.6 mmol, 45%). ^{1}H NMR (400 MHz, $CDCl_3$) δ 3.48 (t, *J* = 7.2 Hz, 2H), 1.52 (m, 2H), 1.20 (m, 10H), 0.77 (t, *J* = 6.4 Hz, 3H) ppm; ^{13}C NMR (100 MHz, $CDCl_3$) δ 159.72, 130.68, 127.88, 38.54, 31.58, 28.92, 28.05, 26.60, 22.43, 13.88.

3. ^{1}H NMR and ^{13}C NMR Spectra

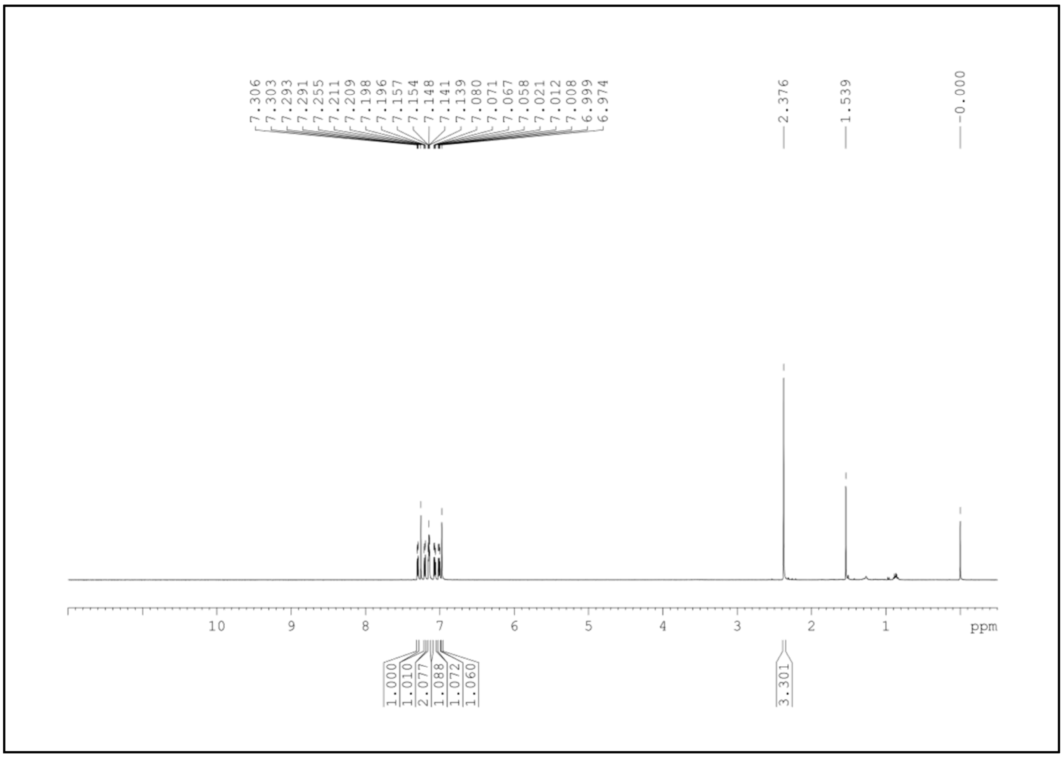

Figure S1. ^{1}H NMR spectrum of Compound S2.

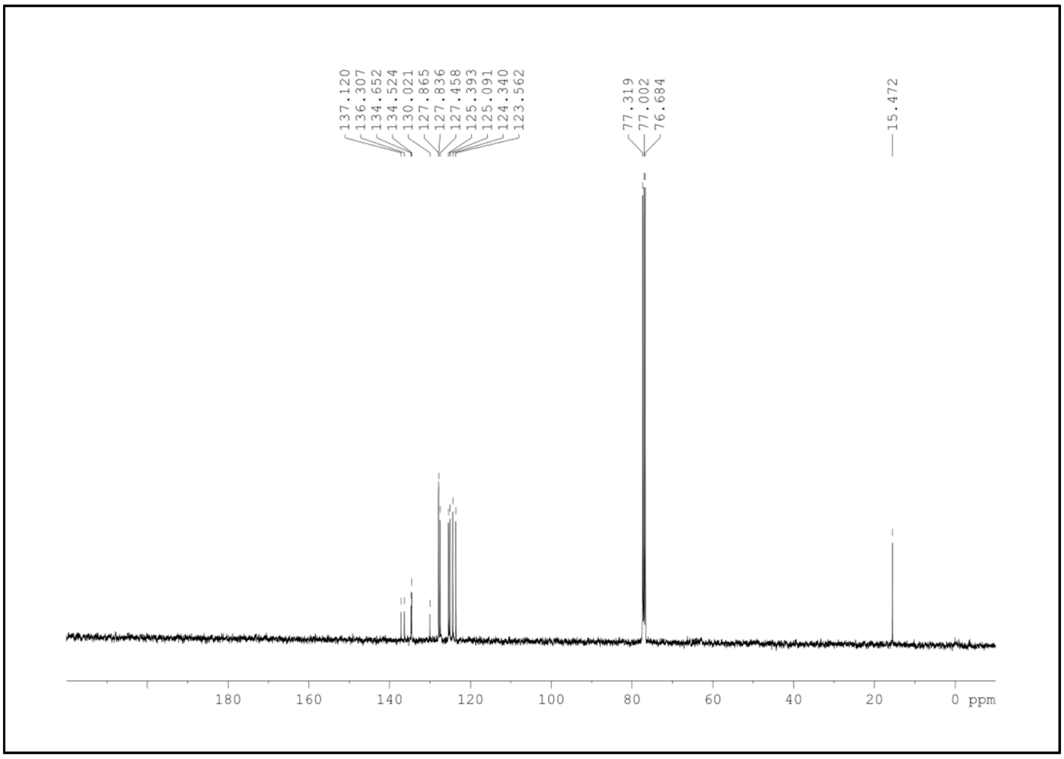

Figure S2. ^{13}C NMR spectrum of Compound S2.

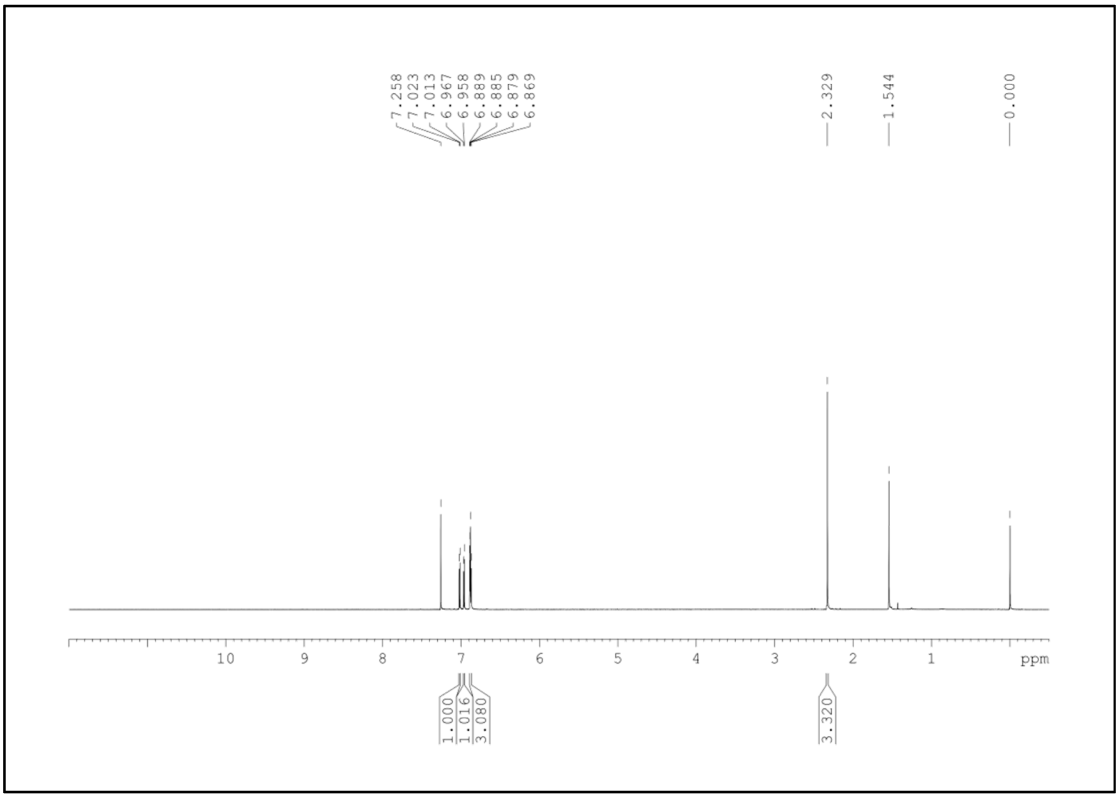

Figure S3. ^{1}H NMR spectrum of Compound S3.

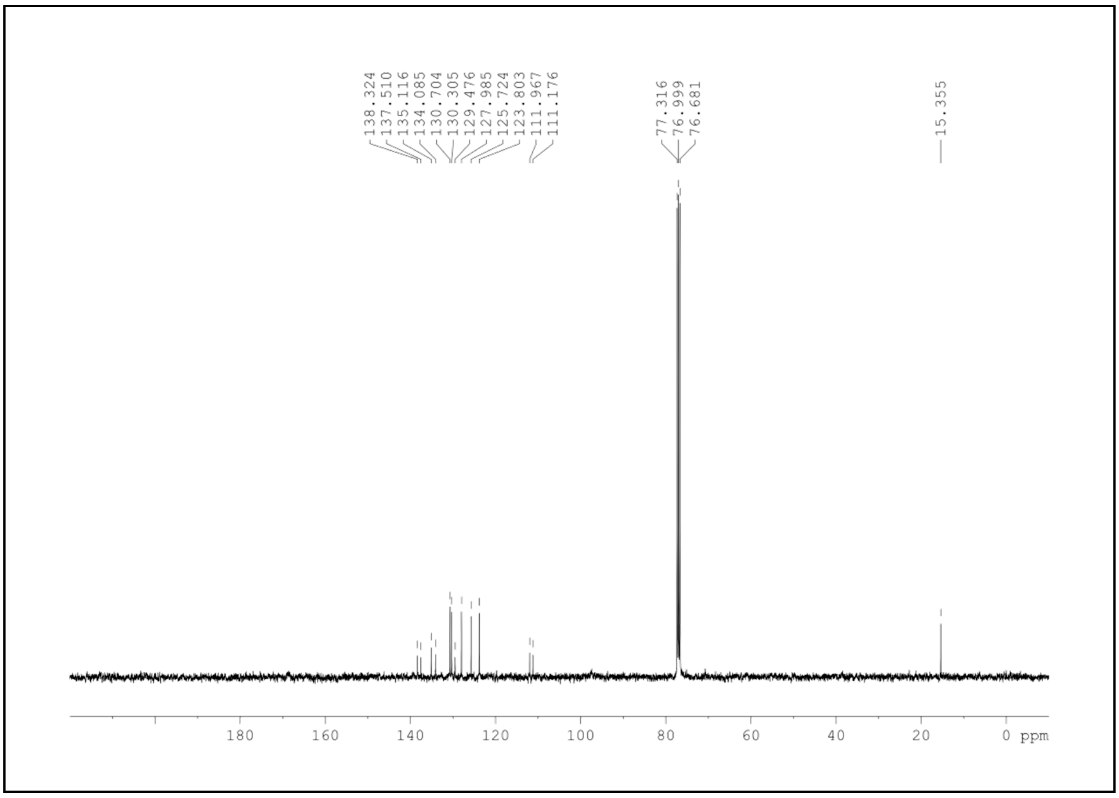

Figure S4. ^{13}C NMR spectrum of Compound S3.

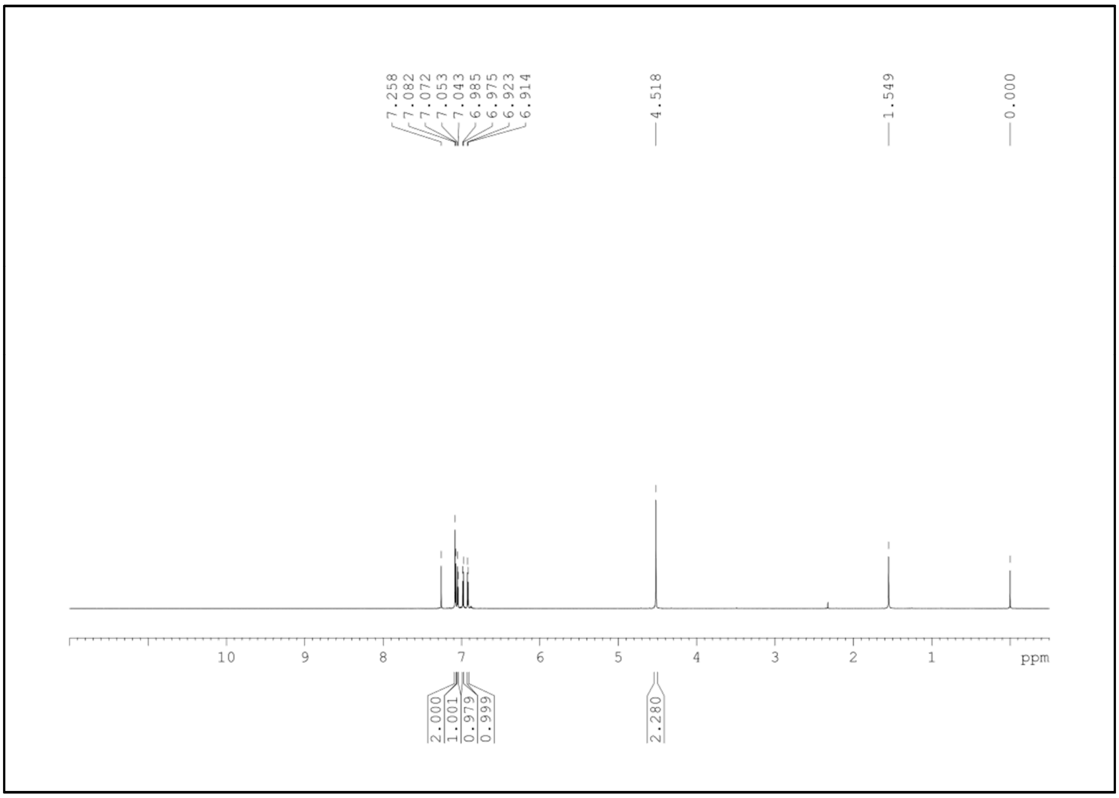

Figure S5. ^{1}H NMR spectrum of Compound S4.

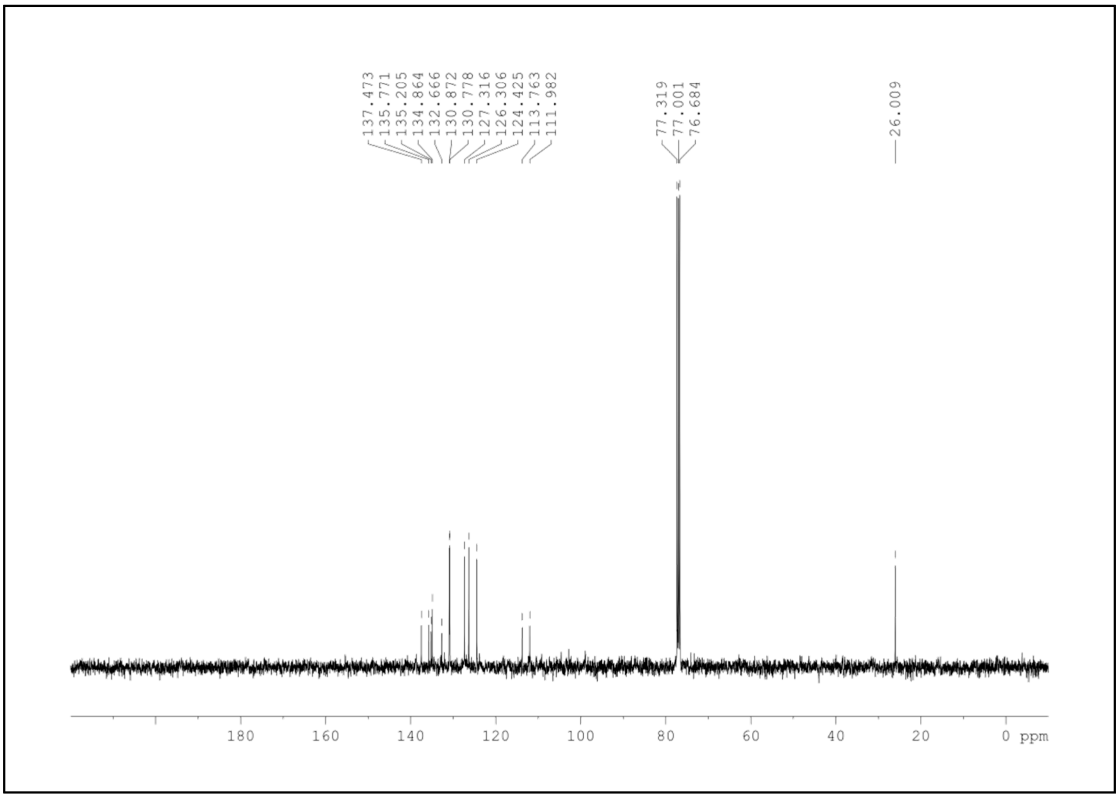

Figure S6. ^{13}C NMR spectrum of Compound S4.

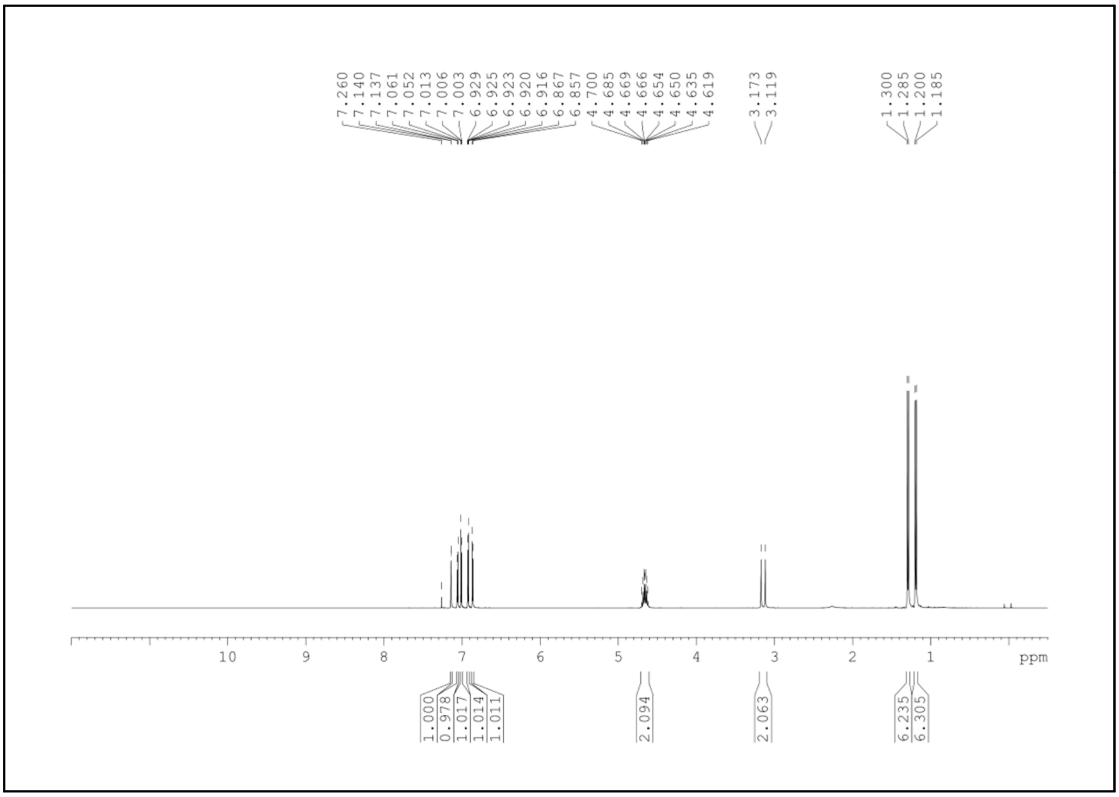

Figure S7. ^{1}H NMR spectrum of Compound 1.

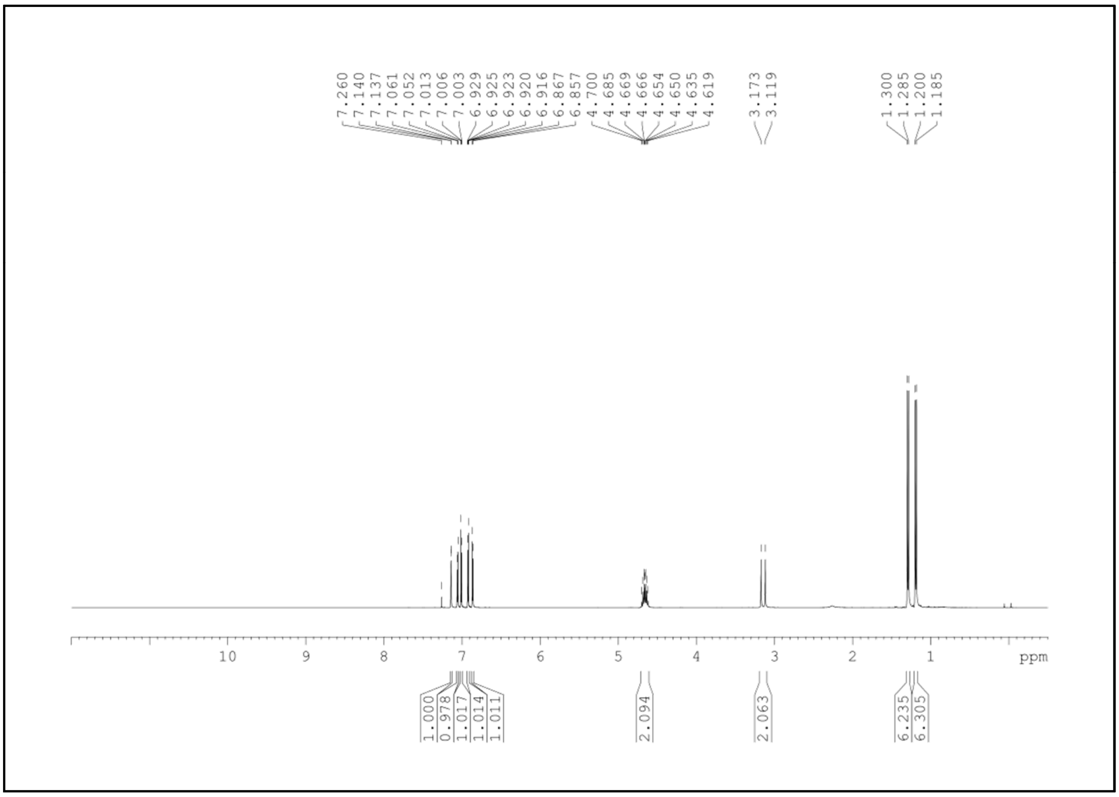

Figure S8. ^{13}C NMR spectrum of Compound 1.

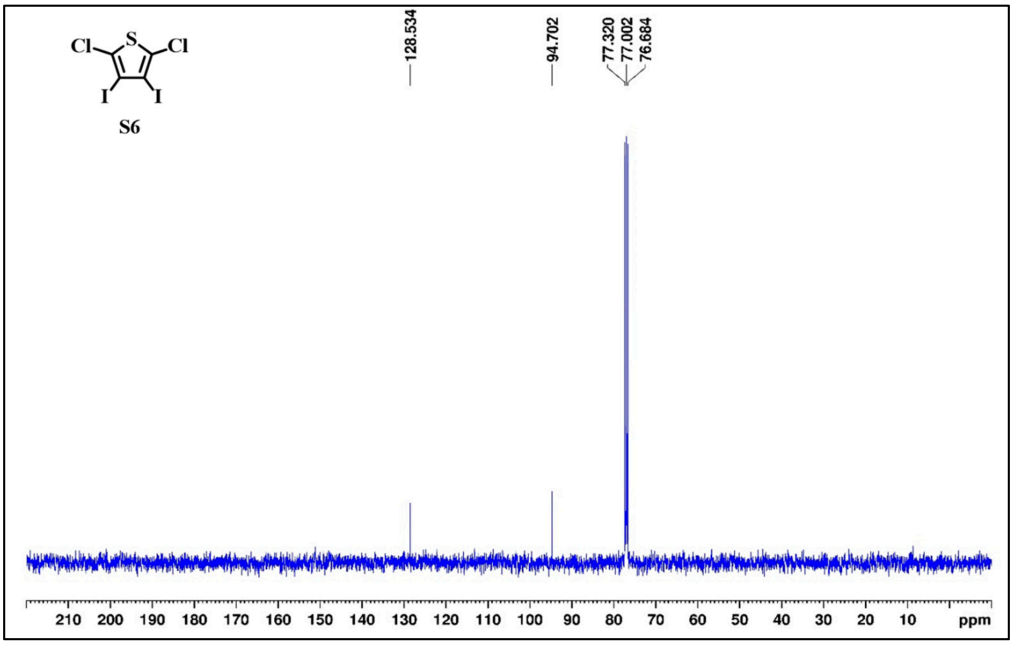

Figure S9. ^{13}C NMR spectrum of Compound S6.

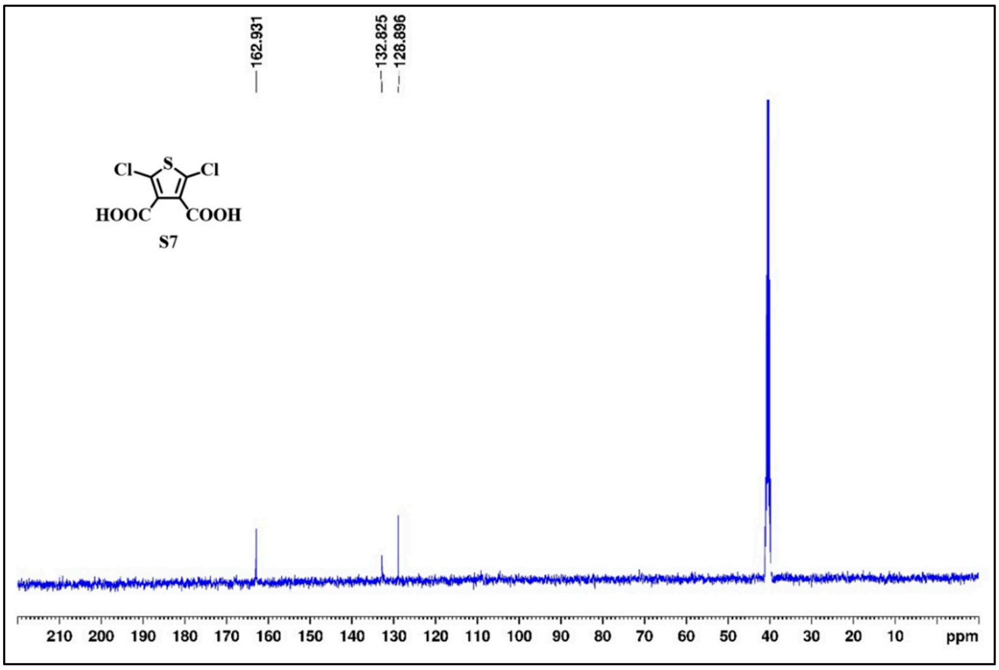

Figure S10. ^{13}C NMR spectrum of Compound S7.

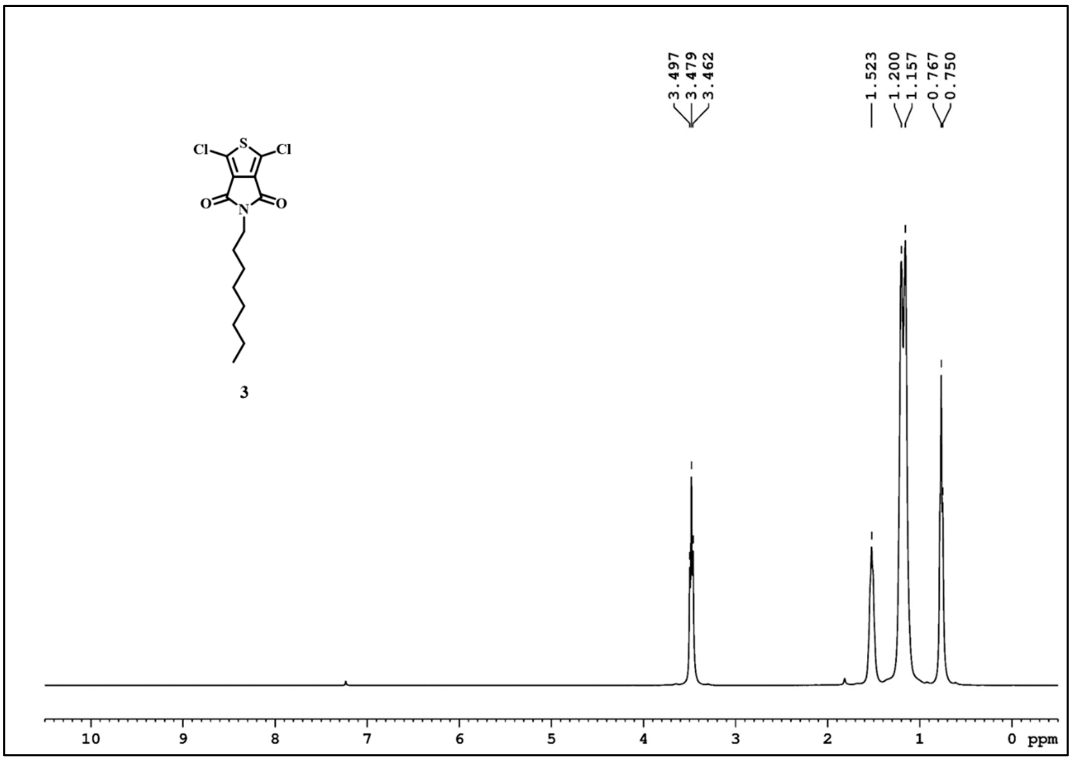

Figure S11. ^{1}H NMR spectrum of Compound 3.

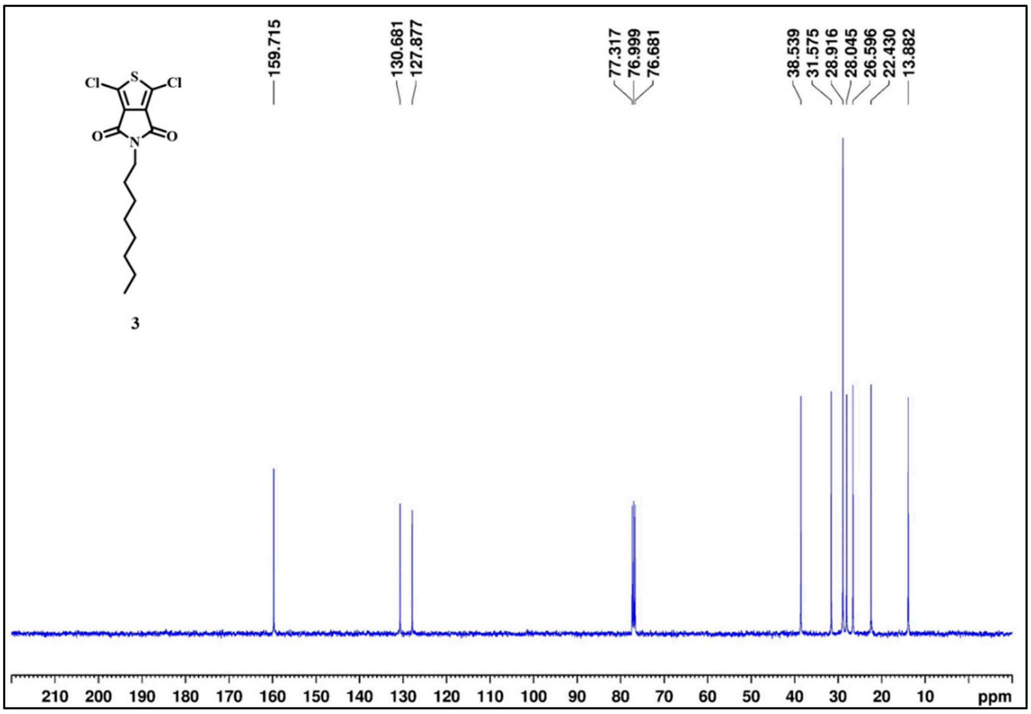

Figure S12. ^{13}C NMR spectrum of Compound 3.

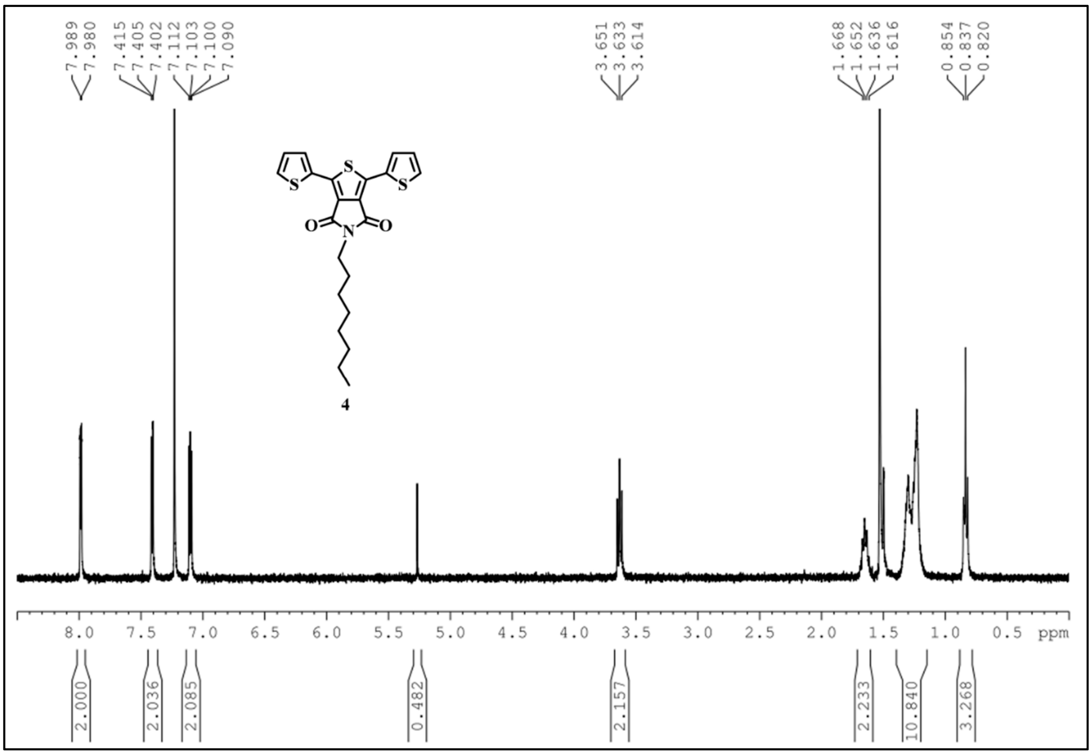

Figure S13. ^{1}H NMR spectrum of Compound 4.

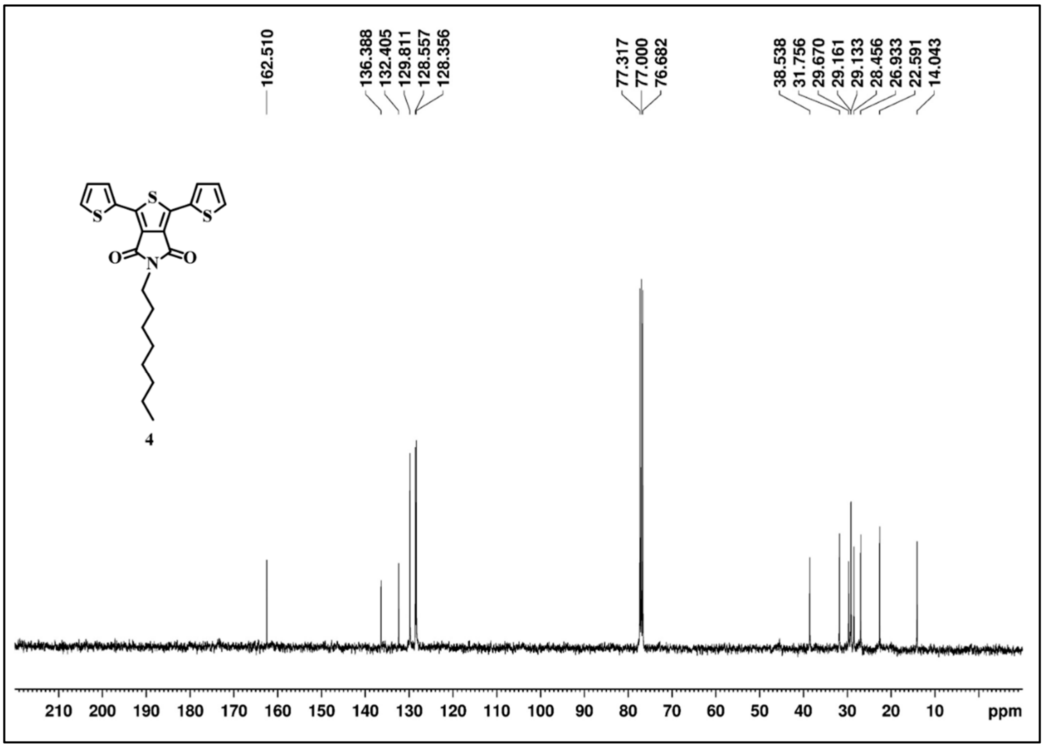

Figure S14. ^{13}C NMR spectrum of Compound 4.

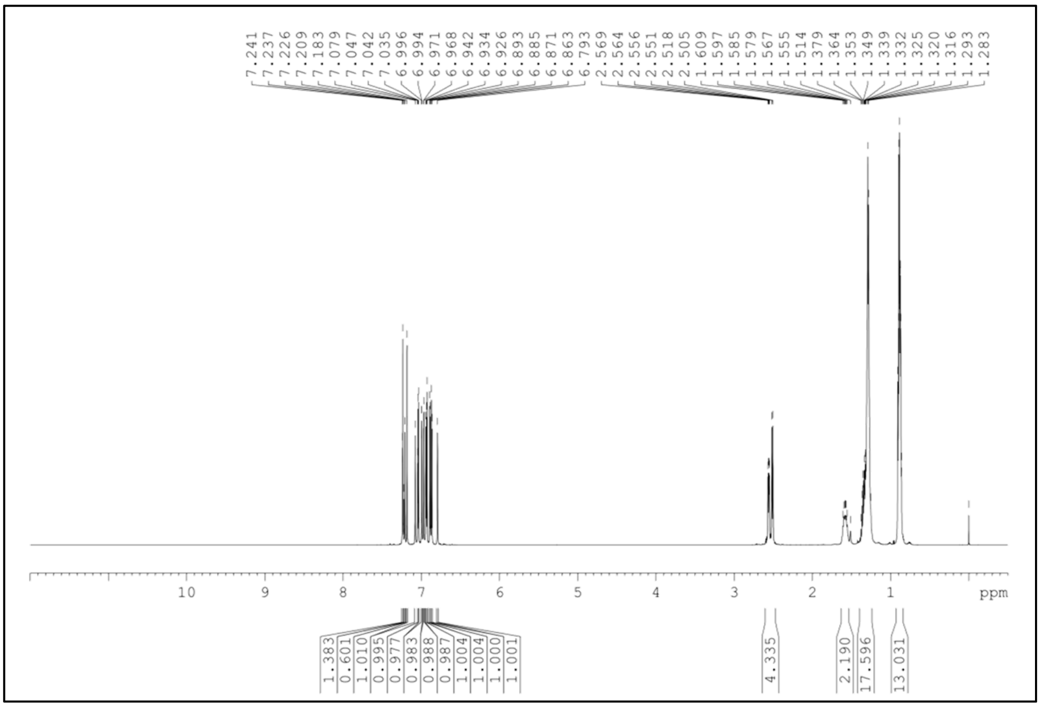

Figure S15. ^{1}H NMR spectrum of Compound M1.

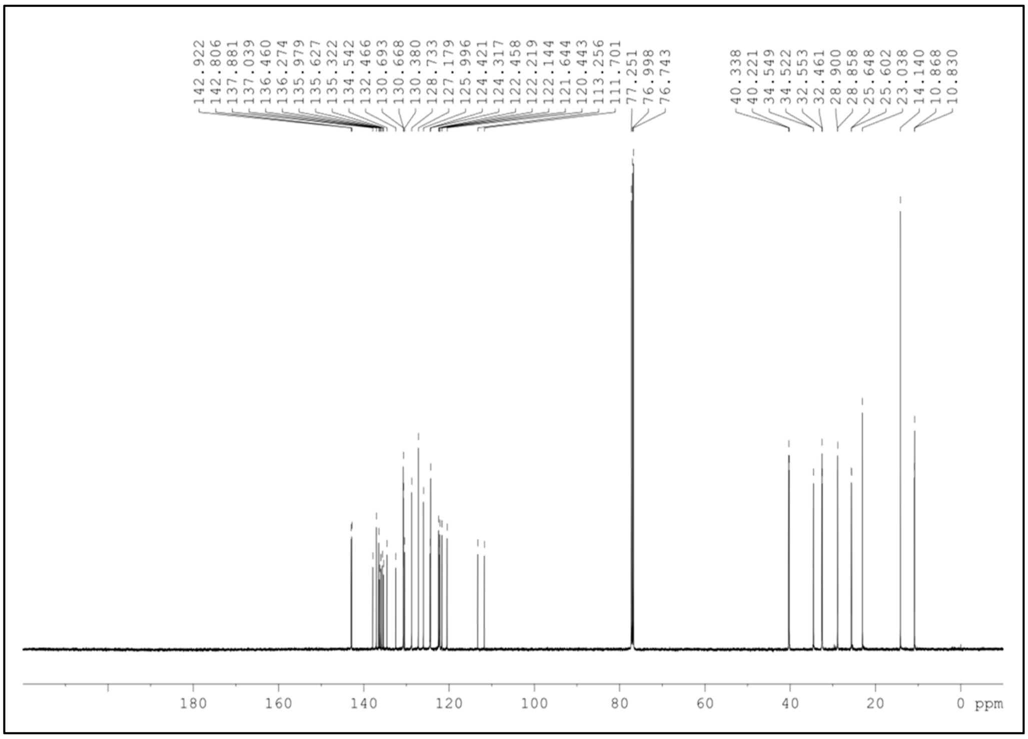

Figure S16. ^{13}C NMR spectrum of Compound M1.

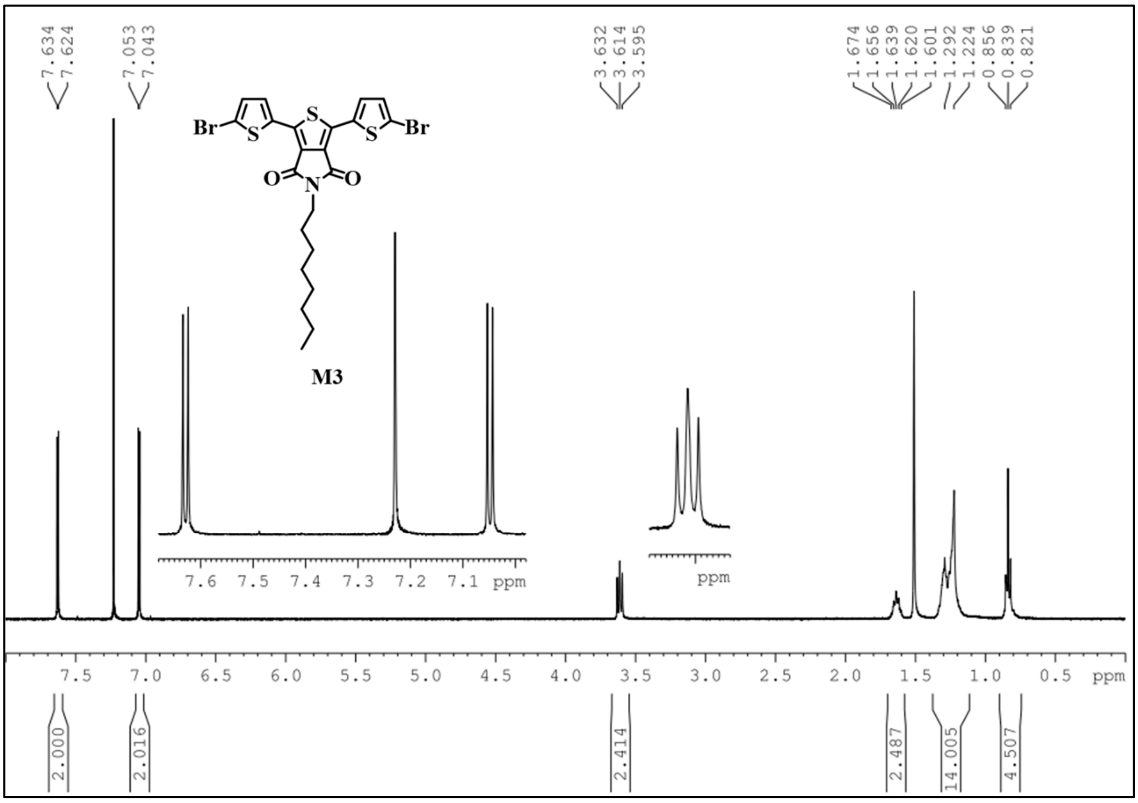

Figure S17. ^{1}H NMR spectrum of Compound M3.

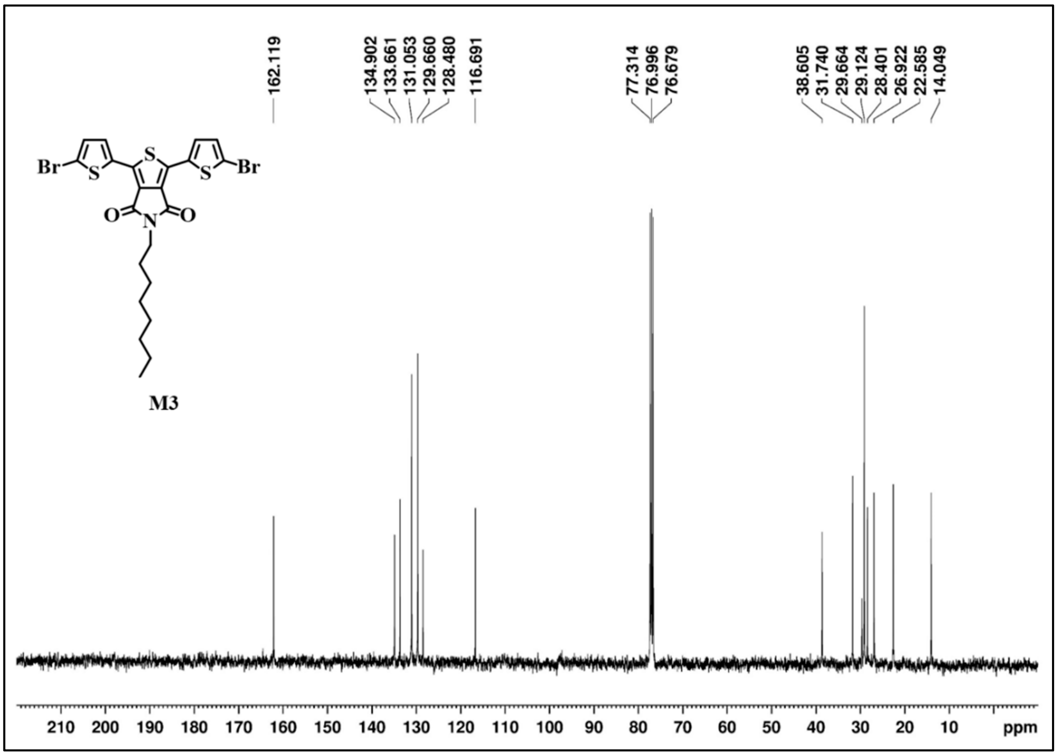

Figure S18. ^{13}C NMR spectrum of Compound M3.

4. TGA and DSC Plots

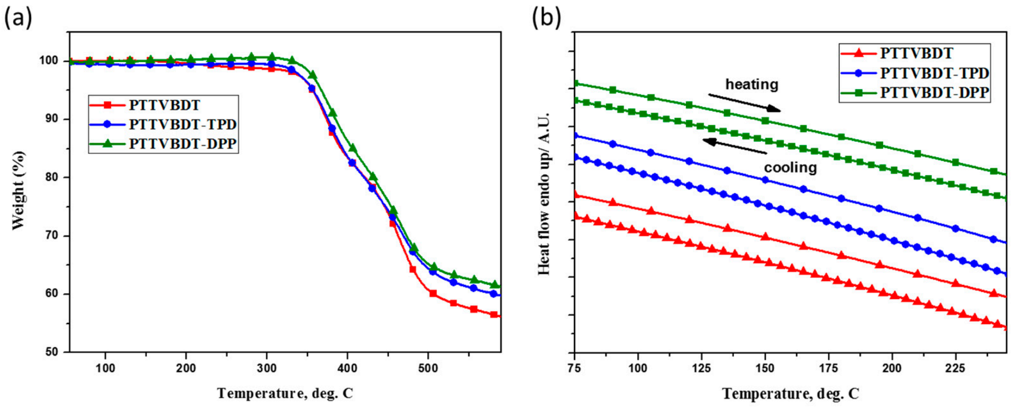

Figure S19. (**a**) TGA and (**b**) DSC second heating profiles of PTTVBDT, PTTVBDT-TPD, and PTTVBDT-DPP with a heating rate of 10 °C/min under N_2 atmosphere and a cooling rate of 10 °C/min.

5. One-Dimensional Grazing Incidence X-ray Diffraction (1D-GIXRD)

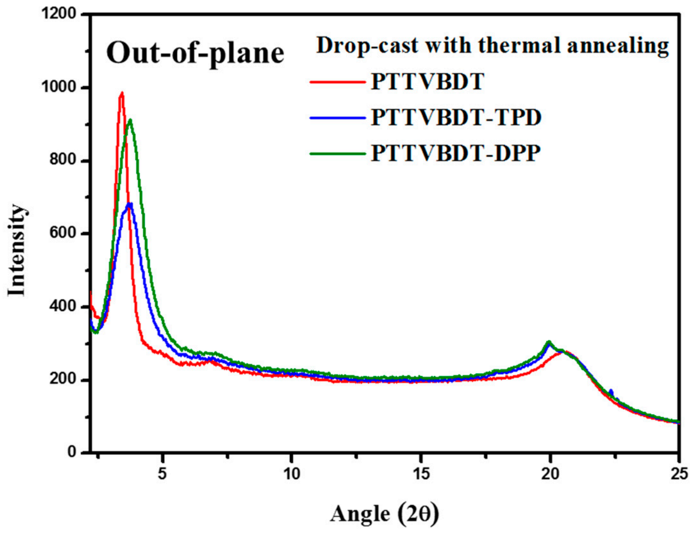

Figure S20. 1D-GIXRD out-of-plane of PTTVBDT, PTTVBDT-TPD, and PTTVBDT-DPP films which were prepared by drop-casting and then thermal annealing at 120 °C, 20 min.

6. Mobility of Polymer with/without $PC_{61}BM$

Table S1. Mobility of PTTVBDT, PTTVBDT-TPD, and PTTVBDT-DPP with/without $PC_{61}BM$ by the SCLC method.

Polymer	Pristine polymer hole mobility ($cm^2·V^{-1}·s^{-1}$)	Blend with $PC_{61}BM$ hole mobility ($cm^2·V^{-1}·s^{-1}$)	Blend with $PC_{61}BM$ electron mobility ($cm^2·V^{-1}·s^{-1}$)	h^+/e^-
PTTVBDT	5.88×10^{-5}	2.01×10^{-5}	3.65×10^{-5}	0.55
PTTVBDT-TPD	6.04×10^{-4}	5.70×10^{-4}	8.37×10^{-4}	0.68
PTTVBDT-DPP	4.63×10^{-4}	4.23×10^{-4}	5.69×10^{-4}	0.74

7. Atomic Force Microscopy (AFM)

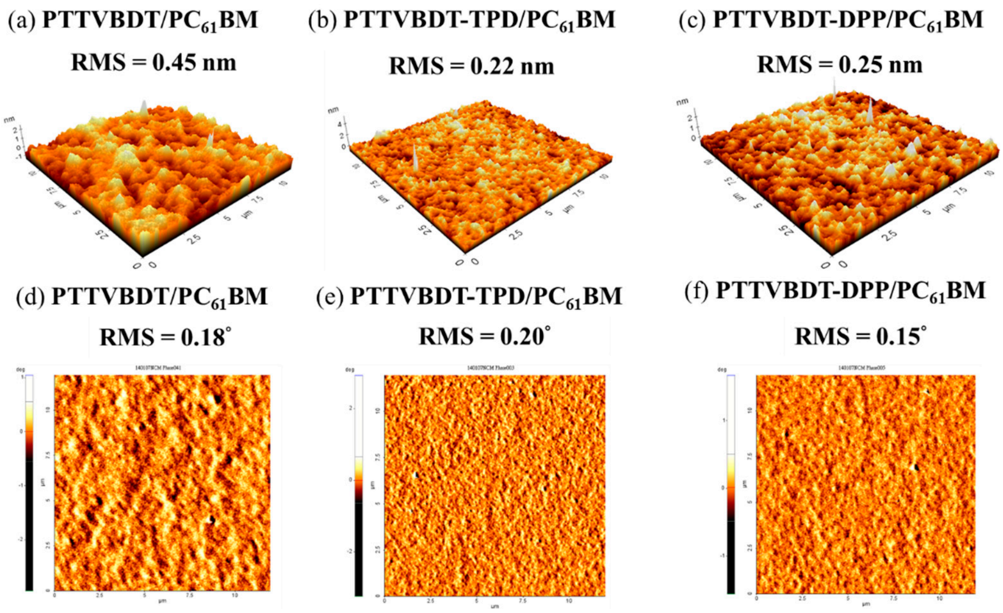

Figure S21. AFM (**a**–**c**) topography images and (**d**–**f**) phase images of spin-coated films of polymer/PC$_{61}$BM blends. The scan sizes for all images are 2 μm × 2 μm.
